# Amelioration of Experimental autoimmune encephalomyelitis and DSS induced colitis by NTG-A-009 through the inhibition of Th1 and Th17 cells differentiation

**DOI:** 10.1038/s41598-018-26088-y

**Published:** 2018-05-17

**Authors:** Suman Acharya, Maheshwor Timilshina, Liyuan Jiang, Sabita Neupane, Dong-Young Choi, Sang Won Park, Sang Yeul Lee, Byeong-Seon Jeong, Jung-Ae Kim, Tae-gyu Nam, Jae-Hoon Chang

**Affiliations:** 1 College of Pharmacy, Yeungnam University, Gyeongsan, 38541 Republic of Korea; 2 Department of Pharmacy and Institute of Pharmaceutical Science and Technology, Hanyang University, Ansan, 15588 Republic of Korea

## Abstract

CD4^+^ T cells are the central for the mammalian adaptive immune system. Naïve CD4^+^ T cells mainly differentiate in to pro-inflammatory Th1, Th2 and Th17 cells upon antigenic stimulation. IFN-γ secreting Th1 cells and IL-17 secreting Th17 cells are found to play key roles in autoimmune diseases like multiple sclerosis (MS) and ulcerative colitis (UC). In this study we found NTG-A-009, 6-aminopyridin-3-ol, has great inhibitory effect on *in vitro* differentiation of Th1 and Th17 cells without affecting regulatory T cells. Moreover, NTG-A-009 had no effect on CD4^+^ T cell proliferation and viability. *In vivo* treatment has shown that NTG-A-009 has ameliorated experimental autoimmune encephalomyelitis (EAE) and dextran sulfate sodium (DSS) induced colitis through the inhibition of Th1 and Th17 cells differentiation. Mechanistically, NTG-A-009 suppressed Th1 and Th17 cells differentiation via the modulation of JAK/STAT signaling pathway. Thus, our data demonstrated that NTG-A-009 ameliorated inflammation through the inhibition of Th1 and Th17 cells generation making it a potential therapeutic candidate for the treatment of inflammatory diseases.

## Introduction

CD4^+^ T cells play crucial role in orchestrating adaptive immune response^1^ which on activation by T cell receptor get differentiated into specific Th lineages like Th1, Th2, Th17 and regulatory T (T_reg_) cells depending upon cytokine milieu of the microenvironment^2,3^. IL-12 induces the differentiation of Th1 cells and predominantly secretes Interferon-γ (IFN-γ) and provides immune response against intracellular pathogens and bacterial infections^4^. Naïve CD4^+^ T cell differentiate into IL-17 producing Th17 cells in the presence of cytokines IL-6 and TGF-β which is actively involved in the clearance of extracellular bacteria and fungi^5^. Although the Th1 and Th17 cells are important for maintaining the immune response, the abnormal activation and differentiation of Th1 and Th17 cells contribute to multiple autoimmune inflammatory diseases^2,4^.

Autoimmune diseases are the conditions wherein the body immune system attacks own tissues afflicting 5–10% of population in the world^5^. Aberrant autoreactive T cell response along with the dysfunction network of the immune system are the key players contributing to human autoimmune disease like multiple sclerosis (MS)^6^. MS is chronic progressive and demyelinating disease of the brain and spinal cord. Auto reactive pathogenic T cells against myelin antigens leads to neurodegeneration and block the impulse conduction at the site of demyelination^7^. Experimental autoimmune encephalomyelitis (EAE) is the extensively studied animal model of MS for more than 40 years^8^. Th1 and Th17 cells produce multiple pro inflammatory cytokines like IFN-γ, IL-17, IL-1β, IL-2 and GM-CSF due to which they can recruit more inflammatory cells into the CNS lesion and are capable of exacerbation of EAE^9^.

Inflammatory bowel disease (IBD) is a chronic inflammatory disorder of the gastrointestinal tract with its two major form, Crohn’s disease (CD) and Ulcerative colitis (UC) whose exact etiology remain unclear^10^. The aberrant differentiation of naïve CD4^+^ T cells in to Th1 and Th17 subsets is major predisposing factors that leads to IBD^11^. UC is primarily associated with the Th1 and Th17 immune response mediated by the overproduction of pro inflammatory cytokines like IFN-γ, IL-1β, TNFα, IL-17 in the colonic mucosa^12–14^. Dextran sulfate sodium (DSS) induced colitis is the most widely studied mouse model with close resemblance to human UC^15^. DSS induced acute colitis model carried out by Alex *et al*. showed that the mice given with DSS for 7 days exhibited increase in IL-12 and IL-17 implying the induction in Th1 and Th17 cells^13^. In other study it has been reported that Th1 and Th17 can independently involve in the pathogenesis of colitis, where it was observed that DSS causes the progressive up regulation of Th1 cytokines like IL-12, IFN-γ, TNF- α^16^. Genome-wide association studies showed that Th17 cells are important for colitis where they recruit and stimulate IL-17A and IL-17F in colonic mucosa^17^.

JAK/STAT signaling pathway is one of the major pathways which regulate Th1 and Th17 cells differentiation^18^. IFN-γ and IL-12 bind with their respective receptors expressed on the surface of naïve CD4^+^ T cells and drive the differentiation of Th1 cell through the activation of signal transducer and activator of transcription 1 (STAT1), STAT4 and T box transcription factor (T-bet)^3^. Likewise, the binding of IL-6 with IL-6 receptor activates retinoid-related orphan receptor γ T (RORγt) and STAT3 and drives Th17 cell differentiation and function^3^. STAT proteins are greatly activated through the phosphorylation of their upstream JAK proteins^19^. JAK/STAT pathway has dragged significant attention as a therapeutic target in autoimmune diseases and inflammation^20^.

Several diseases modifying treatment for MS and UC including FTY720 (fingolimod), natalizumab (tysabri), azathioprine and cyclosporine have been commonly used^21,22^. However, these treatments still have side effects, response failure and marginal efficacy. So, there is the dire need for the search of new therapeutic agent which can inhibit inflammatory diseases without having inhibitory effect to other immune cells. Structurally, NTG-A-009 (6-aminopyridin-3-ol) is the derivatives of aminopyridinol compounds having the feature of thiadiazole heterocyclic moiety. Various aminopyridinol derivatives have shown diverse biological activities with strong antiangiogenic and anti-colitis activities^23,24^. Thus, we initially examined the effect of NTG-A-009 on Th1 and Th17 cells differentiation and we found that NTG-A-009 exhibited strong inhibitory effect on Th1 and Th17 cells differentiation without effect on regulatory T cells. This result encouraged us to find therapeutic potential of NTG-A-009 on autoimmune diseases caused by Th1 and Th17 cells. *In vivo* study revealed that NTG-A-009 treatment prevented the onset of EAE and alleviates on going EAE by reducing the generation of Th1 and Th17 cells in EAE mice. Furthermore, NTG-A-009 treatment was effective in attenuating DSS induced clinical manifestations, histological damage and colon shortening by showing inhibitory effect on pro inflammatory responses of Th1 and Th17 cells. Mechanistically, NTG-A-009 reduced the differentiation of naïve CD4^+^ T cells by inhibiting phosphorylation of JAK1 and JAK2 and its downstream STAT1 and STAT4 in Th1 cell and STAT3 in Th17 cell. We compared NTG-A-009 with commercial JAK inhibitor, tofacitinib, and corticosteroid triamcinolone, which have potent anti-inflammatory properties. In contrast to tofacitinib and triamcinolone, NTG-A-009 did not affect the activation, proliferation and viability of CD4^+^ T cells. Thus, our findings suggest that NTG-A-009 is relatively safe in terms of cell toxicity and can be used as novel potential therapeutic agent for the treatment of Th1 and Th17 mediated inflammation and autoimmune diseases through the modulation of JAK/STAT signaling pathway.

## Results

### NTG-A-009 inhibits Th1 and Th17 cells differentiation *in vitro*

**Figure 1:**
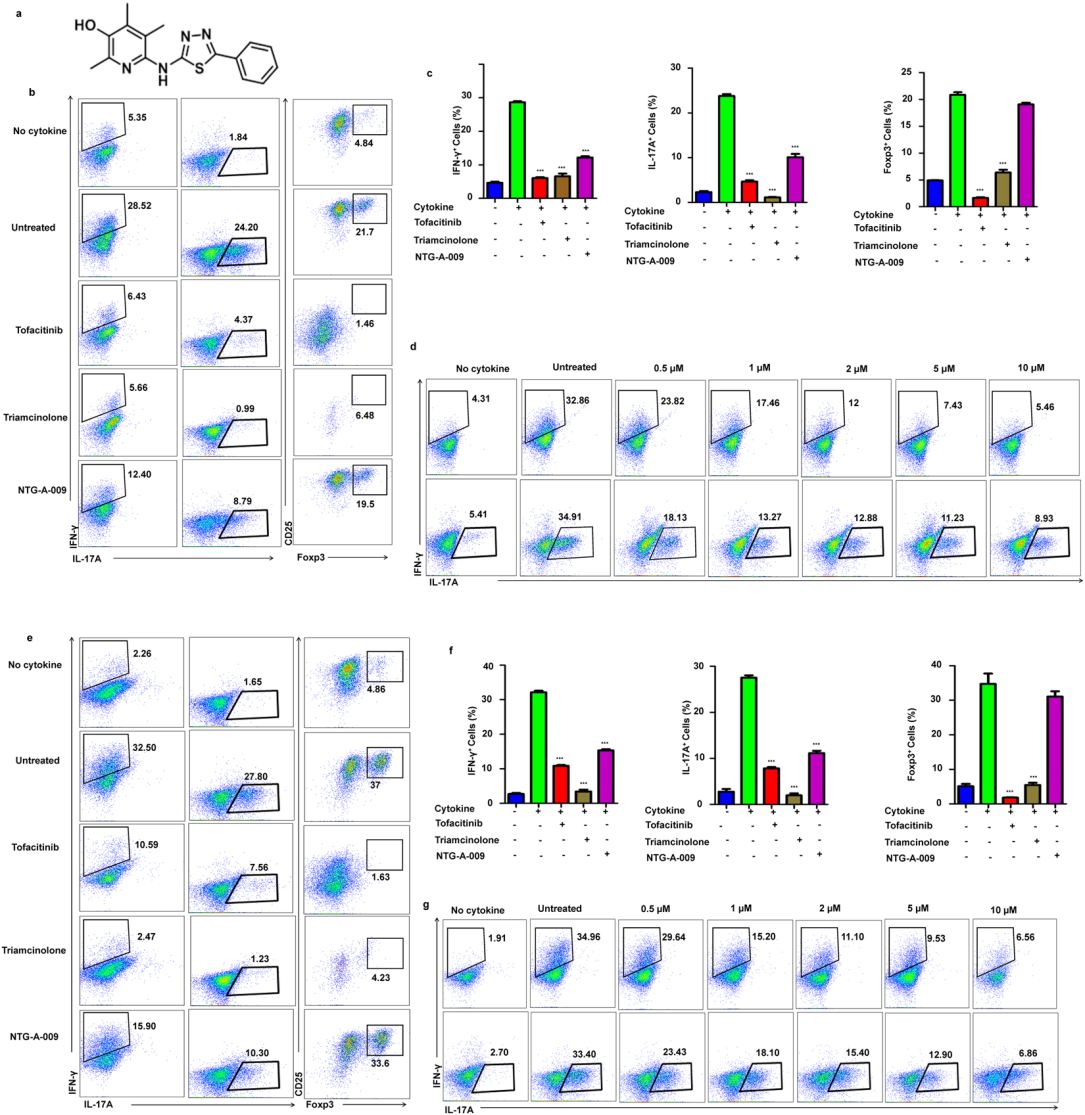
NTG-A-009 reduces Th1, Th17 cells differentiation *in vitro*. Naïve CD4^+^ T cells isolated from spleen and lymph nodes were stimulated with anti-CD3 (1 μg/ml) and anti-CD28 (1 μg/ml) under Th1, Th17 and T_reg_ inducing conditions with NTG-A-009 (1 μM), Tofacitinib (1 μM) or Triamcinolone (1 µM). (**a**) Chemical structure of NTG-A-009. (**b,c**) The percentage of IFN-γ^+^ Th1, IL-17A^+^ Th17 and FoxP3^+^ T_reg_ cells was determined by flow cytometry. (**d**) Different concentration of NTG-A-009 was used to find its effect in Th1 and Th17 cells in anti-CD3 antibody coating system. (**e,f**) The antigen specific differentiation of Th1, Th17 and T reg cells was done by the isolation of CD4^+^ T cells and irradiated antigen presenting cells from 6–10 weeks OTII mice and stimulated under Th1, Th17 and T_reg_ polarizing conditions in the presence of OVA _323–339_ (0.1 μM) and incubated for 72 hour for Th1 and Th17 and 96 hours for T_reg_ cells differentiation. Cells were then restimulated with PMA, ionomycin and golgistop and analyzed by FACS through intracellular staining. (**g**) Different doses of NTG-A-009 were cultured under Th1, Th17 condition with the cells from OT-II transgenic mice. Data were quantified in bar diagram. Data represent three independent experiments. Mean ± SEM of the triplicates are shown. ***P < *0.001*.

Th1 and Th17 cells are main players contributing to organ specific autoimmune disease and inflammation^25–27^. The chemical compound used in this study was NTG-A-009 which is 6-aminopyridin-3-ol derivative (Fig. 1a). In order to assess the effect of this compound on differentiation of T cell, we stimulated naïve CD4^+^ T cells isolated from spleen and lymph node with anti-CD3 and anti-CD28 in Th differentiation conditions. NTG-A-009 (1 µM) inhibited the differentiation of Th1 and Th17 cells although the commercial agents tofacitinib (1 µM) and triamcinolone (1 µM) have great inhibitory effect on Th1 and Th17 cells differentiation (Fig. 1b). In contrast, NTG-A-009 did not inhibit the differentiation of T_reg_ cells while tofacitinib and triamcinolone profoundly inhibited the T_reg_ cell activation and differentiation (Fig. 1b,c). Furthermore NTG-A-009 reduced Th1 and Th17 cell differentiation in a dose dependent manner (Fig. 1d). These data collectively demonstrate that NTG-A-009 inhibited *in vitro* Th1 and Th17 cells differentiation as similar to commercially available agents tofacitinib and triamcinolone.

To examine whether NTG-A-009 has inhibitory effect on antigen specific Th1 and Th17 cells differentiation, we used OT-II mice which have the transgenic expression of an ova-specific T- cell antigen receptor. OT-II CD4^+^ T cells have high expression of transgenic αβ- T cell receptors which are specific for chicken ovalbumin (OVA_323–339_) in the context of I-A b and is expressed in 80% to 90% of T cells^28^. Due to high proportion of CD4^+^ T cells expressing TCR, OTII transgenic mice is useful for the induction and responsiveness of T cells to ova antigens^29^. So the use of CD4^+^ T cells from these mice best explains the effect of NTG-A-009 on antigen specific T cells. Naïve CD4^+^ T cells isolated from spleen and lymph nodes of OT-II mice were cultured with NTG-A-009, tofacitinib or triamcinolone in the presence of ova peptide and irradiated antigen presenting cells (APC) for 72 hours. Consistent with Fig. 1b,c, NTG-A-009 inhibited the differentiation of Th1 and Th17 cells but had no effect on regulatory T cells (Fig. 1e,f). Next, to assess the effect of NTG-A-009 on Th1 and Th17 cells differentiation in dose dependent manner, CD4^+^ T cells isolated from OT-II mice were stimulated with ova peptide in the presence of different concentration of NTG-A-009. The differentiation of Th1 and Th17 cells were decreased in dose dependent manner (Fig. 1g). These findings suggest that NTG-A-009 inhibited the differentiation of antigen specific T cells.

The potential of NTG-A-009 for inhibition of *in vitro* Th1 and Th17 differentiation prompted us to examine whether this compound inhibit inflammation induced by highly activated T cells *in vivo*, mice were intraperitoneally administered with the mixture of OVA (2 mg/ml) and complete Freund’s adjuvant (CFA). NTG-A-009 (2 mg/kg/day) was then administered through intraperitoneal route in every other day for 6 days and mice were sacrificed on day 7. Induction of inflammation was indicated by increase in the size of spleen but in the mice treated with NTG-A-009 had significant decrease in the spleen size (Supplementary Fig. S1a). We next analyzed Th1 and Th17 cell generation in spleen and lymph node from NTG-A-009 treated or untreated mice. CFA/OVA injection causes the strong induction of ova specific Th1 and Th17 cells and treatment with NTG-A-009 reduced the generation of Th1 and Th17 cells in the spleen and lymph nodes of CFA/OVA treated group (Supplementary Fig. S1b). Furthermore, the amount of pro-inflammatory cytokines IFN-γ and IL-17A produced by CD4^+^ T cells was critically decreased in NTG-A-009 treated mice (Fig. S1c). The overall data suggest that NTG-A-009 inhibits the generation of IFN-γ specific Th1 cells and IL-17A specific Th17 cells *in vivo*.

### NTG-A-009 has no negative effect on T cells proliferation and viability

**Figure 2:**
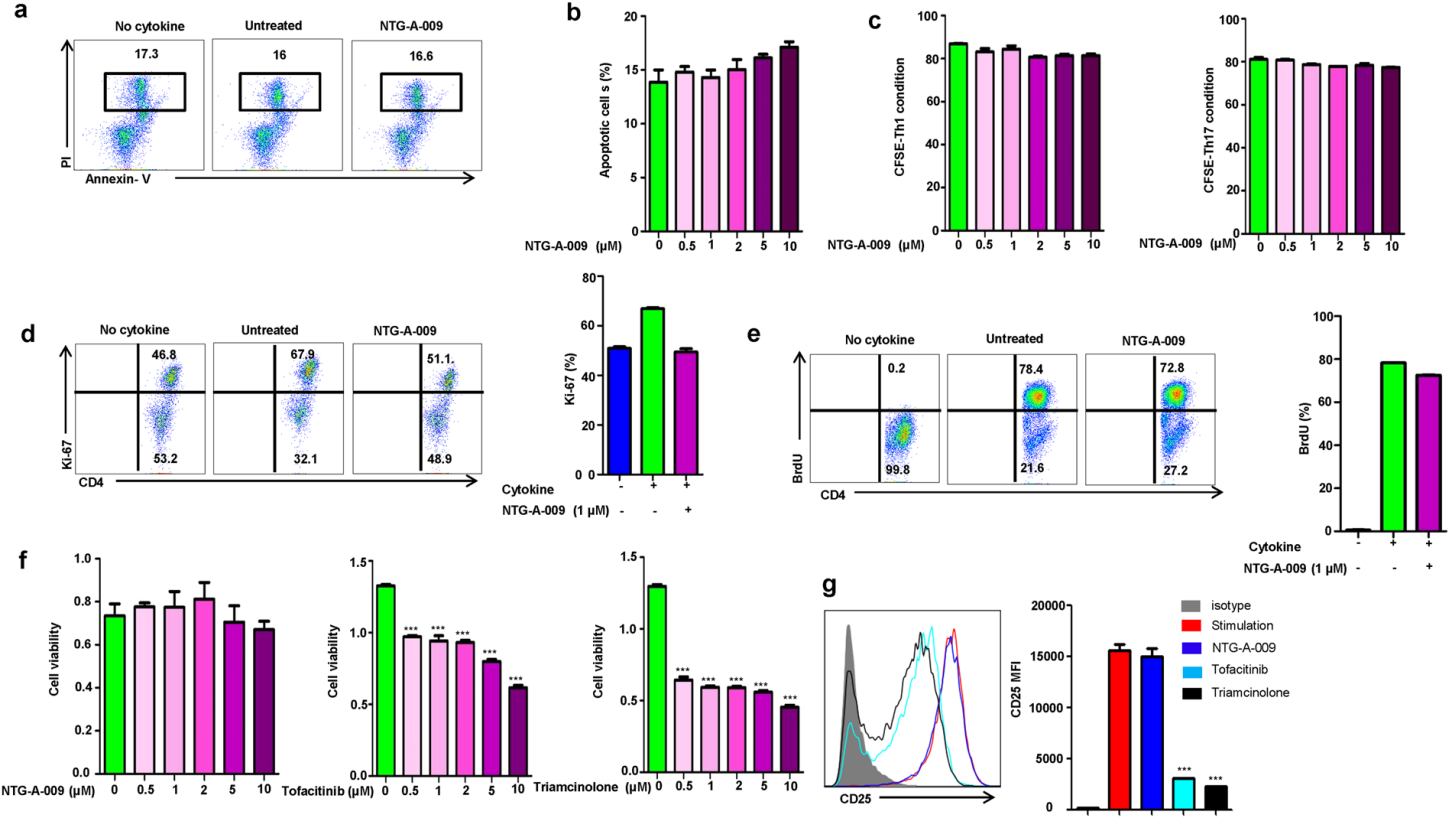
Effect of NTG-A-009 on CD4^+^ T cell proliferation, viability and apoptosis. (**a**) Naïve CD4^+^ T cells isolated from spleen and lymph nodes were cultured with or without NTG-A-009 (1 μM) under Th1 condition for 72 hour and percentage of live cells were detected by Annexin-V and PI staining and analyzed by flow cytometry. (**b**) Apoptotic cells percentage was analyzed by treating with the different doses of NTG-A-009. (**c**) Naïve CD4^+^ T cells labelled with CFSE were activated with anti-CD3 and anti-CD28 under Th1 and Th17 conditions with the different doses of NTG-A-009. Cell proliferation was assessed by CFSE dilution by flow cytometry. (**d**) CD4^+^ T cell proliferation detection by the analysis of Ki-67 protein expression under Th1 condition with or without NTG-A-009. Bar diagram represent the percentage of Ki-67 positive CD4^+^ T cells. (**e**) Naïve CD4^+^ T cells and APCs were isolated from spleen and lymph node and stimulated under Th1 condition in the presence of BrdU (10 μM) with or without NTG-A-009. (**f**) Splenic lymphocytes were isolated from normal C57BL/6 mice and incubated with the different concentrations of NTG-A-009, Tofacitinib and Triamcinolone and Cell viability was measured by MTS assay. (**g**) CD4^+^ T cells isolated from spleen and lymph nodes were stimulated with anti-CD3 (1 μg/ml) and anti-CD28 (1 μg/ml) for 24 hours in the presence of NTG-A-009 (1 μM), tofacitinib (1 μM) or triamcinolone (1 µM). The expression of CD25 was determined by flow cytometry. Data are the representative of three independent experiments. ***P < 0.001.

To find whether inhibitory effects of NTG-A-009 are mediated by the cytotoxicity of the compound, CD4^+^ T cells were stimulated with anti-CD3, anti-CD28 and cytokines in the presence or absence of NTG-A-009 for 3 days. After 3 days of activation, cells were stained with Annexin V and PI. The percentage of apoptotic cells was not significantly high with the untreated group even at the concentration of NTG-A-009 (10 µM) suggesting that inhibitory effect of NTG-A-009 is not due to apoptosis (Fig. 2a,b). Next, we analyzed the role of NTG-A-009 on CD4^+^ T cell proliferation. CD4^+^ T cells were labeled with CFSE and stimulated with anti-CD3, anti-CD28 and cultured with different doses of NTG-A-009 under Th1 and Th17 conditions. No considerable decrease in the proliferation of Th1 and Th17 cells was seen with NTG-A-009 treatment (Fig. 2c). Ki-67 which is a proliferation marker was analyzed after anti-CD3, anti-CD28 and cytokine stimulation. Only marginal decrease in Ki-67 expression was seen after treatment with NTG-A-009 (Fig. 2d). Furthermore, *in vitro* proliferation measured by thymidine analog bromodeoxyuridine (BrdU) labeling demonstrated that NTG-A-009 did not inhibit the proliferation of activated T cells under Th1-polarizing condition (Fig. 2e). Moreover, we examined the toxicity of NTG-A-009 with tofacitinib and triamcinolone by MTS (3-(4, 5-dimethylthiazol-2-yl)-5-(3-carboxymethoxyphenyl)-2-(4-sulfophenyl)-2H-tetrazolium) assay (Fig. 2f). Tofacitinib is the first JAK inhibitor and found potent for the treatment of rhemutoid arthritis^30^. Triamcinolone is synthetic corticosteroid with antiangiogenic and anti-inflammatory activities^31^. In our study cell viability assay had demonstrated that tofacitinib and triamcinolone had cytotoxic effects on CD4^+^ T cells although this effect was more pronounced with triamcinolone treatment (Fig. 2f). We found that the treatment with NTG-A-009 had no effect on cell viability which conferred the safety of NTG-A-009 in terms of cytotoxicity (Fig. 2f). Furthermore, NTG-A-009 had no effect on T cell activation marker CD25 in comparison to tofacitinib and triamcinolone (Fig. 2g). The overall results suggest that NTG-A-009 had no effect on normal T cell proliferation, activation and does not induce cytotoxicity.

### NTG-A-009 reduces the differentiation of Th1 and Th17 through the modulation of JAK/STAT pathway

**Figure 3:**
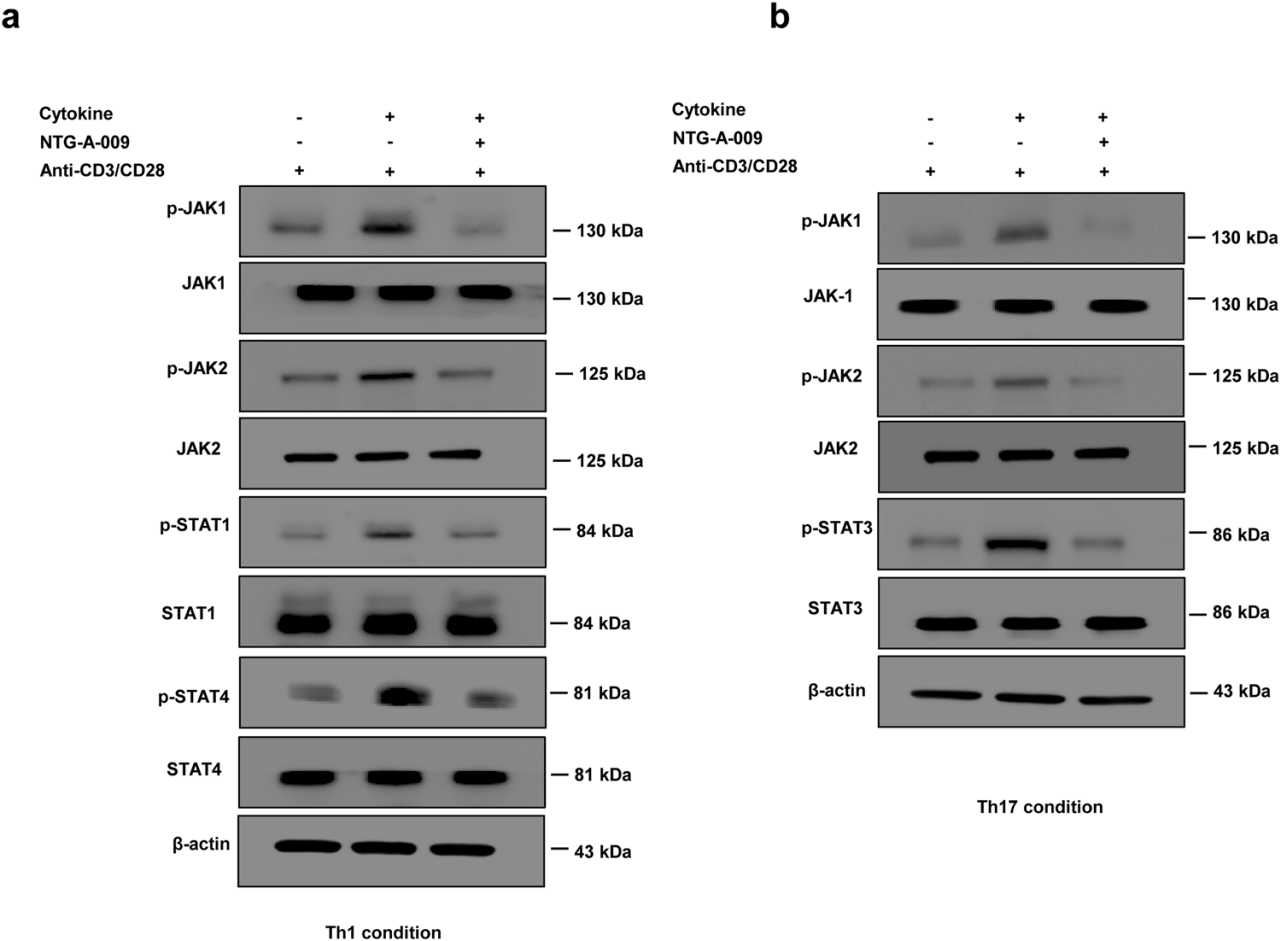
NTG-A-009 reduces Th1 and Th17 cells differentiation through the inhibition of JAK/STAT pathway. (**a**) Naïve CD4^+^ T cells isolated from spleen and lympnodes were cultured with or without NTG-A-009 under Th1 condition and re stimulated with anti-CD3 (1 μg/ml) and anti-CD28(1 μg/ml) followed by the analysis of the expression of indicated proteins in JAK/STAT pathway by western blot. Phosphorylated and total form of STAT1, STAT4, and JAK1/2 were analyzed by immunoblotting under Th1 condition. (**b**) Phosphorylated and total form of STAT3 and JAK1/2 were detected by immunoblotting under Th17 condition. Full length blots and gels were shown in supplementary information- 7, 8, 9 and 10. Data are the representative of three independent experiments.

We next attempted to find the signaling pathway that mediates the effect of NTG-A-009 on Th1 and Th17 cell differentiation. JAK/STAT pathway is one of the major signaling pathway that regulates the differentiation and functions of Th1 and Th17 cells^32^. We hypothesized that NTG-A-009 might have regulatory effects on Th1 and Th17 differentiation via the inhibition of JAK/STAT pathway. For this, we isolated splenic T cells and stimulated with anti-CD3, anti-CD28 under Th1 and Th17 conditions and analyzed for JAK/STAT proteins by western blotting. STAT1 was activated by autocrine IFN-γ whereas; STAT4 is mainly activated by IL-12 both of which are important for *in vitro* differentiation of Th1 cells^33,34^. IL-6, IL-21 and IL-23 activate STAT3 which is important for differentiation, functions and amplification of Th17 cells^35,36^. NTG-A-009 decreased the phosphorylation of STAT1 and STAT4 (Fig. 3a). Similarly, Th17 differentiation was also markedly inhibited by NTG-A-009, which was indicated by decreased phosphorylation of STAT3 (Fig. 3b). JAK family including JAK-1, JAK-2, JAK-3 and Tyk-2 are essential for signaling pathways of different cytokines which are implicated in the pathogenesis of several autoimmune diseases^19^. So, we also checked for JAK protein which is the upstream for STAT. The expression of phosphorylated JAK1/2 was considerably decreased by NTG-A-009 which was consistent with the reduction of Th1 and Th17 cells (Fig. 3a,b). Our finding demonstrated that the treatment with NTG-A-009 decreases the phosphorylation of JAK1/JAK2, p-STAT1/4 in Th1 and p-STAT3 in Th17 without having any changes on total form of JAK and STAT proteins. To demonstrate the effect of NTG-A-009 on overexpressed STAT1 and STAT3, we isolate the CD4^+^ T cells and transfected with STAT1 (STAT1 alpha Y701F pRc/CMV, addgene plasmid) and STAT3 (pcDNA3-STAT3-Y705F, addgene plasmid) expressing plasmid respectively. Immunoblotting result showed that STAT1 and STAT3 were overexpressed in transfected group as compared to non-transfected or control vector transfected group (Supplementary Fig. 5a). We also found that overexpression of STAT1 or STAT3 has recovered the decreased phosphorylation of STAT1 and STAT3 even after the NTG-A-009 treatment (Supplementary Fig. 5a). This result suggests that NTG-A-009 mediated inhibition of the STAT1 and STAT3 phosphorylation is recovered by STAT1 and STAT3 overexpression.

Furthermore to see whether the overexpression of STAT1 and STAT3 could reverse the inhibition of Th1 and Th17 differentiation, we perform *in vitro* Th1 and Th17 differentiation of CD4^+^ T cells from STAT1, STAT3 transfected and non-transfected group with or without NTG-A-009. We found that the differentiation of Th1 was significantly higher in STAT1 overexpressed cells and overexpression of the STAT1 significantly recovered the *in vitro* Th1 differentiation even after NTG-A-009 treatment (Supplementary Fig. 6a). Again the differentiation of Th17 cells was significantly higher in STAT3 overexpressed cells and overexpression of the STAT3 significantly recovered the *in vitro* Th17 differentiation after NTG-A-009 treatment (Supplementary Fig. 6b). Altogether, these results clearly suggest that NTG-A-009 inhibits the Th1 and Th17 differentiation by regulating the JAK/STAT signaling pathway.

Thus, we conclude that NTG-A-009 reduces Th1 and Th17 cells differentiation through the inhibition of JAK/STAT signaling pathway (Supplementary Fig. S4).

### NTG-A-009 ameliorates EAE by reducing inflammatory T cells

**Figure 4:**
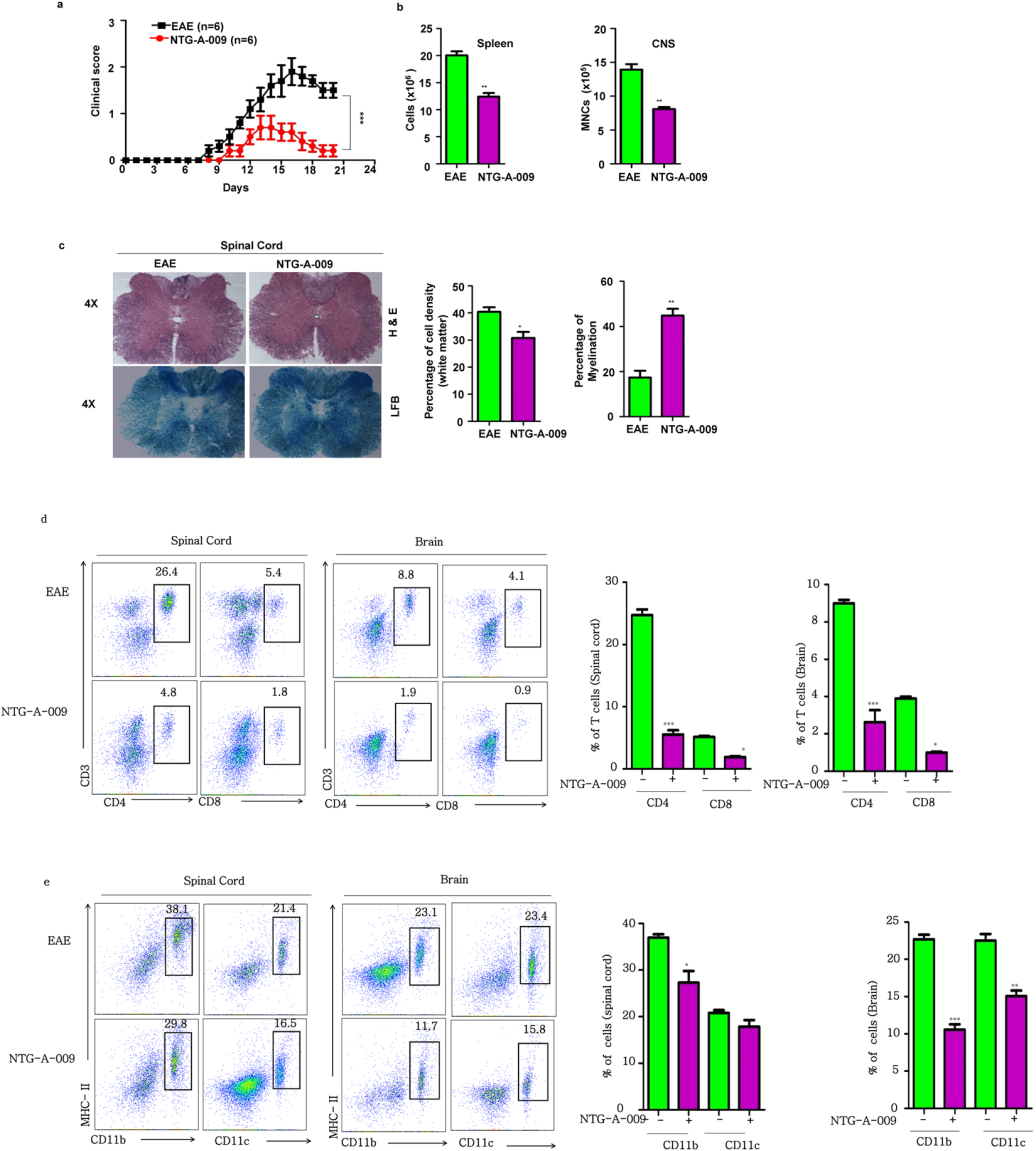
Induction, amelioration and histological analysis of EAE. Active EAE was induced in C57BL/6 female mice (8–12 weeks) by MOG_35–55_. NTG-A-009 (2 mg/kg/day) or vehicle control was administered subcutaneously from the day 1 post immunization as described in materials and methods. (**a**) The disease severity was analyzed on the basis of sign and symptoms. Clinical score in the mice was recorded in daily basis. (**b**) Total no. of splenocytes and mononuclear cells (MNCs) obtained from EAE and NTG-A-009 treated mice. (**c**) Sections of spinal cord obtained from NTG-A-009 and EAE mice at day 21post immunization were analyzed for degree of inflammation by H&E (magnification 4X) which was quantified by analyzing the percentage of cell density in white matter. Luxol fast blue staining (magnification 4X) of spinal cord sections for analyzing the degree of demyelination and was quantified by the total cell density in white matter. (**d**) At day 21, total mononuclear cells were obtained from brain and spinal cord of EAE and NTG-A-009 treated mice. The percentage of CD4^+^ and CD8^+^ T cells was analyzed by flow cytometry. (**e**) The percentage CD11b^+^ and CD11c^+^ cells in MNCs derived from brain and spinal cord was analyzed by flow cytometry. Mean ± SEM of the triplicates are shown. Data represent three independent experiments. *P < 0.05. **P < *0.01*; ***P < *0.001* was determined by student’s *t-* test or two-way ANOVA.

We found that NTG-A-009 reduced the differentiation of Th1 and Th17 cells *in vitro* and reduced CFA-OVA induced inflammation by inhibiting IFN-γ and IL-17A *in vivo* (Supplementary Fig. S1). This finding prompted us to investigate the effect of NTG-A-009 on Th1 and Th17 mediated autoimmune disease. To this end, we chose EAE model, a well-established mice model for MS. EAE was induced in mice by the immunization of MOG_35–55_ peptide in CFA and pertussis toxin as explained in treatment protocol. NTG-A-009 (2 mg/kg/day) was administered in every other day intraperitoneally. As shown in Fig. 4a, typical course of EAE was developed in mice which were characterized by disease onset at day 8. Treatment with NTG-A-009 resulted in significant reduction in disease severity indicated by decreasing clinical scores (Fig. 4a). The therapeutic efficacy of NTG-A-009 was further aided by significant reduction in the number of CNS infiltrating MNCs (mononuclear cells) and splenocytes (Fig. 4b). Furthermore to evaluate the role of NTG-A-009 in the treatment of EAE, we isolated brain and spinal cord from EAE and NTG-A-009 treated mice and performed histopathological analysis. Comparison to EAE mice, NTG-A-009 treatment markedly reduced the inflammation and showed improved myelination as indicated by H&E and LFB staining respectively (Fig. 4c). Similarly, decreased in infiltrating cell was seen in brain of NTG-A-009 treated mice (Supplementary Fig. S2). Next we analyzed CD4^+^ T cells and CD8^+^ T cells infiltration in brain and spinal cord. There was high infiltration of CD4^+^ T cells in EAE mice which was obviously decreased in spinal cord and brain of NTG-A-009 treated mice (Fig. 4d). Similarly, CD8^+^ T cells infiltration was also decreased with NTG-A-009 treatment (Fig. 4d). Antigen presenting cell (APCs) in CNS participates in creating self-destructive environment through the secretion of inflammatory factors or by the presentation of myelin epitopes to autoreactive T cells thereby aiding the EAE progression^37^. We found increase in the CD11b^+^ macrophages/microglia and CD11c^+^ dendritic cells in brain and spinal cord of EAE mice but this pattern was decreased with the treatment of NTG-A-009 (Fig. 4e) however, very less or almost no effect was observed in the CD11c^+^ dendritic cells in the spinal cord with NTG-A-009 treatment. These findings suggest that CD4^+^, CD8^+^ T cells along with the APCs in CNS is critical for the pathogenesis of EAE which was profoundly decreased after NTG-A-009 treatment.

**Figure 5:**
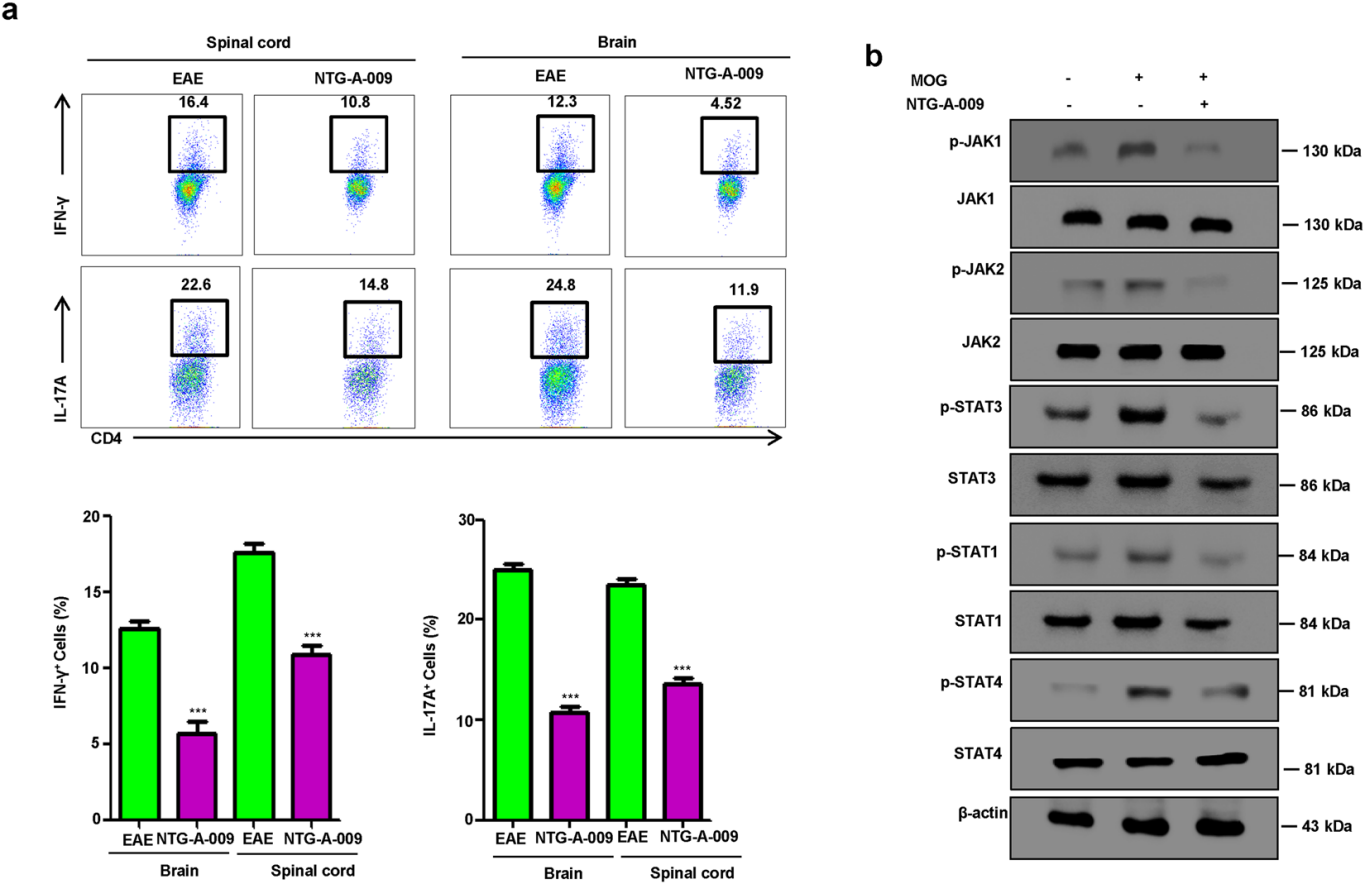
EAE amelioration by NTG-A-009 through the reduction of Th1 and Th17 cells via the modulation of JAK/STAT pathway. (**a**) The percentage of Th1 and Th17 cells was analyzed by FACS via the intracellular staining of IFN-γ and IL-17A through the cells obtained from the brain and spinal cord of EAE and NTG-A-009 treated mice at day 21 post immunization. (**b**) Splenocytes derived from the EAE and NTG-A-009 treated mice were further overnight restimulated with MOG_35–55_ peptide and CD4^+^ T cells were analyzed. Immunoblotting was done for the expression of phosphorylated form of JAK/STAT proteins in JAJK/STAT pathway. Full length blots and gels were shown in supplementary information-6. Data represent three independent experiments.

It has been previously studied that autoreactive Th1 and Th17 cells are critically involved in the pathogenesis of MS and its mice model, EAE^38–41^. We checked for the role of NTG-A-009 on Th1 and Th17 cells generation by intracellular staining of MNCs derived from brain and spinal cord. Consistent with *in vitro* and *in vivo* findings there was significant reduction in IFN-γ specific Th1 and IL-17 specific Th17 cells in NTG-A-009 treated group (Fig. 5a). Regarding the molecular mechanism behind the amelioration of EAE, with the treatment of NTG-A-009 there was decreased expression of phosphorylated JAK1/2, p-STAT1/p-STAT4 for Th1 cells and p-STAT3 for Th17 cells (Fig. 5b). Collectively these findings indicate that NTG-A-009 ameliorate EAE in mice by reducing Th1 and Th17 cells infiltration in CNS through the inhibition of JAK/STAT pathway.

### NTG-A-009 attenuates colitis in DSS treated mice

**Figure 6:**
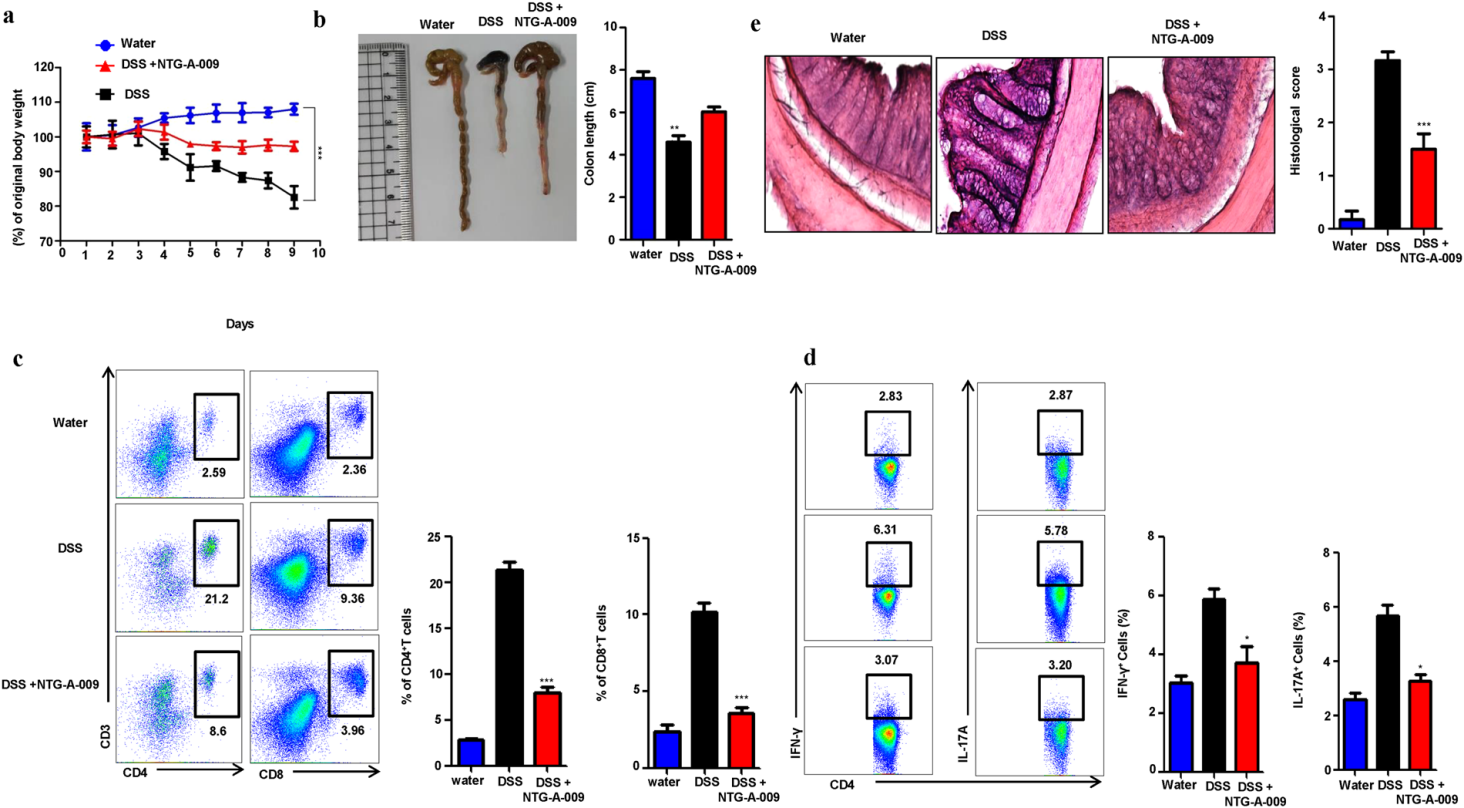
NTG-A-009 inhibits Th1 and Th17 cells *in vivo* and attenuate DSS induced colitis. C57BL/6 mice were orally administered with 2.5% (w/v) DSS in drinking water for 7 days to induce colitis. NTG-A-009 was orally administered every day (n = 6 mice in each group). (**a**) Body weight of water, DSS and NTG-A-009 treated mice was recorded daily and presented as percentage of original body weight. (**b**) Colon length of mice treated with water, DSS and NTG-A-009. (**c**) CD4^+^ and CD8^+^ T cells infiltration was detected from the Lymphocytes isolated from colonic lamina propria of water, DSS and NTG-A-009 treated group and analyzed by FACS. (**d**) The percentage of Th1 and Th17 cells was determined by the stimulation of splenocytes from each group with PMA, ionomycin and golgistop for 4 hour and analyzed by FACS through intracellular staining of IFN-γ and IL-17. (**e**) H&E staining of colonic section with histological score (magnification 20X). Data represent three independent experiments. Two-way anova (A) or *P < 0.05, **P < 0.01, ***P < 0.001.

Dextran sulfate sodium (DSS) induced colitis is widely used mouse model for human inflammatory diseases (IBD)^42^ and characterized by increased epithelial injury and inflammatory cytokines production by inflammatory cells^43,44^. Studies have suggested that mouse given with DSS for 7 days showed increased IL-12 and IL-17 suggesting an induction of Th1 and Th17 cells^10,13^. So to evaluate the therapeutic efficacy of NTG-A-009 in colitis, we carried DSS induced colitis model in mice. Mice were orally administered with 2.5% DSS in drinking water and NTG-A-009 (2 mg/kg/day) was orally administered every day while untreated group received only water. DSS treated mice showed clear manifestation of colitis including rectal bleeding, loss of body weight and shortening of colon in comparison to only water treated group (Fig. 6a,b) while these symptoms were significantly suppressed in NTG-A-009 treated group (Fig. 6a,b). Lymphocytes were isolated from colonic lamina propira and tested for CD4^+^ and CD8^+^ T cells infiltration. NTG-A-009 reduced infiltration of T cells (Fig. 6c). Furthermore, we analyzed the effect of NTG-A-009 on generation of Th1 and Th17 cells. We isolated CD4^+^ T cells from spleen and stimulated with PMA, ionomycin and golgistop for 4 hour and stained for IFN-γ and IL-17A and analyzed by FACS. In comparison to DSS treated group, there was reduced percentage of Th1 and Th17 cells generation in NTG-A-009 treated group (Fig. 6d). We next observed the histological changes in colon by H&E staining. Only water treated mice had normal colonic histology with intact epithelium and no infiltration of inflammatory cells (Fig. 6e). DSS administered mice displayed injury and acute colitis along with massive mucosal ulceration, crypt damage and severe inflammation while NTG-A-009 treatment significantly ameliorated DSS induced histological damages of colon tissues indicated by decreased histological score (Fig. 6e). These findings suggest that NTG-A-009 promotes the recovery of DSS induced colitis by exerting inhibitory effects on inflammatory responses showed by T cells. Taken together, these data demonstrate that NTG-A-009 has a therapeutic efficacy against UC.

## Discussion

Naïve CD4^+^ T cells after TCR and cytokine stimulation get differentiated in to effector subsets Th1, Th2, Th17 and Treg cells which are important in protection and repairing tissues after infections^11^. For the induction of autoimmune diseases the combined effect of Th1 and Th17 cells is important^45^. Therefore, investigating novel compound which specifically target Th1 and Th17 cells is of great clinical significance. Our screening campaign using diverse chemicals revealed NTG-A-009 to be the most potent compound with anti-inflammatory activity. In this study we found that NTG-A-009 can regulate the differentiation of Th1 and Th17 cells without having inhibitory effect on regulatory T cells. A part from the adaptive immune cells, innate immune cells mediate inflammation by recognizing microbes and other danger signals and provide first line of defense^46^. They are also found to play important role in autoimmune diseases like MS^47^. We found that NTG-A-009 had no effects on mRNA level of different cytokines of BMDC stimulated by LPS (Fig. S3). On the basis of these findings we can believe that NTG-A-009 has potential to regulate inflammatory T cells without broad effect on T cell biology. Adjuvants act by targeting antigens to APCs thereby enhancing antigen presentation by MHC^48^. Presentation of antigens along with cytokine and co-stimulatory molecules drives the differentiation of naïve CD4^+^ T cells in effector cells^49^. So, we employed OVA based inflammatory model in mice, in which OVA was combined with CFA (complete Freund’s adjuvant) and induces powerful OVA specific Th1 and Th17 inflammatory immune response. NTG-A-009 inhibited CFA-OVA induced inflammation by negatively regulating IFN-γ^+^ Th1 and IL-17A^+^ Th17 cells which further strengthen the efficacy of NTG-A-009 in reducing inflammation *in vivo*.

Activation of T cells is important for the proliferation and differentiation in to effector cells^50^. In the presence of TCR and costimulatory signaling, T cells proliferate and produce different cytokines that can activate T cells. T cells expresses activation markers like CD25 when they are activated^51^. Tofacitinib and triamcinolone, suppressed CD25 expression after TCR stimulation unlike NTG-A-009 (Fig. 2g). From this observation, we can assume that tofacitinib and triamcinolone might regulate TCR signaling but NTG-A-009 does not. Thus, NTG-A-009 treatment had no effect on early activation, proliferation and apoptosis. In the literature, it has been found that tofacitinib inhibits the proliferation and viability of T cells^20,52^. Consistent with these findings, we demonstrated that tofacitinib inhibits the viability of CD4^+^ T cells in dose dependent manner. Triamcinolone is anti-inflammatory steroid which have therapeutic significance for the treatment of various inflammatory autoimmune diseases^53^. It has been reported that triamcinolone decreases the viability of human lens epithelial cells through apoptotic pathway^54^. To the best of our knowledge this is the first study to explore the toxicity of triamcinolone on T cells which may suggest the judicial and discriminate use of triamcinolone for the treatment of autoimmune diseases. Reduced in the differentiation of Th1 and Th17 cells with triamcinolone treatment may be due to its cytotoxic effect. Such inhibitory and toxic effect was not seen with NTG-A-009 treatment which attributes its safety and therapeutic potential. Thus, this study could provide idea that NTG-A-009 could be alternative of conventionally used drug for the treatment of inflammatory diseases however exhaustive study regarding the same is required in forthcoming days.

Multiple sclerosis (MS) is the chronic demyelinating autoimmune disease of the central nervous system and is the prototypic Th1 and Th17 mediated autoimmune disorders^55^. IFN-γ secreting Th1 cells^56^ and IL-17 secreting Th17 cells^57^ are first primed in the periphery after then migrate in to CNS causing demyelination and axonal loss and subsequent neurological disability^40^. Th1 and Th17 cells further exacerbate the condition by recruiting other inflammatory cells in CNS via the productions of other pro inflammatory cytokines including IFN-γ, IL-17A, IL-1β, IL-2 and GM-CSF^9^. Hence, the compounds which target to inflammatory Th1 and Th17 cells are of imminent need to manage autoimmune disorders like MS. Our *in vitro* and *in vivo* data showed that NTG-A-009 suppresses the generation of Th1 and Th17 cells and attenuated EAE. NTG-A-009 significantly reduced CD4^+^ and CD8^+^ T cells infiltration in brain and spinal cord at the peak of the EAE inflammation. The reduction in inflammation and improvement in myelination in CNS by NTG-A-009 treatment is due to decreased generation of Th1 and Th17 cells. These findings reinforce our understanding of beneficial effect of NTG-A-009 for the management of autoimmune diseases which help to develop the effective therapeutic approach to combat T cell mediated autoimmune response.

Antigen presenting cells like macrophages, microglia, and astrocytes play an important role in the pathogenesis and progression of EAE by presenting myelin epitopes to autoreactive T cells^37,58^. IFN-γ and IL-17 enhance the expression of MHC-II, macrophage activation and trafficking of leukocyte in to CNS^59–61^. We found the Low recruitment of microglia and macrophages in CNS after NTG-A-009 treatment however we did not show the direct relation between the low recruitment of microglia and macrophage with reduced Th1 and Th17 cells. These result demonstrated that NTG-A-009 ameliorate EAE through the inhibition of Th1 and Th17 cells generation in CNS.

The treatment of NTG-A-009 did not show the suppression of CD25, an activation marker. This support us to assume that NTG-A-009 regulates the cytokine signaling but not TCR signaling. JAK/STAT pathway is principle signaling mechanism where almost 40 cytokines receptors signal through this signaling system^62^. Studies have shown that different cytokines like IFN-γ, IL-12, IL-17A, and IL-23 are involved in pathogenesis of EAE through JAK/STAT signaling pathway^63,64^. The activation of STAT1 occurs by IFN-γ during *in vitro* Th1 differentiation^34^ while IL-12 activates STAT4 for Th1 differentiation through the activation of its upstream protein JAK2^65^. Th17 differentiation requires IL-6/IL-23 to activate STAT3 through JAK1/2 activation^66^. So, the inhibition of JAK/STAT pathway by NTG-A-009 attributed to significant reduction in Th1 and Th17 cells differentiation. It is also worth noting that NTG-A-009 critically decreases the phosphorylation of JAK1 than its JAK2 counterparts. Studies have showed that JAK1/STAT3 mediate the pathway which contribute to the resistance to cancer targeted drug^67^. The extensive inhibition of JAK1 also may offer favorable clinical efficacy of NTG-A-009 in the treatment of cancer.

We found that NTG-A-009 inhibited Th1 and Th17 cells *in vitro* and *in vivo* condition and ameliorated inflammation induced by CFA/OVA administration. Th1 and Th17 cells are main culprit for the pathogenesis of EAE. Therefore, to check the clinical efficacy of NTG-A-009 on Th1 and Th17 induced autoimmune diseases we chose EAE model, which is the commonly used experimental model for human multiple sclerosis in mice. It has been shown that JAK/STAT proteins are actively involved in EAE models^64^. STAT4 deficient mice are defective in Th1 cell generation and failed to induce EAE^68^. Likewise, there is defect in generation of Th17 in STAT3 deficient mice and are resistant to EAE^69^. The STAT proteins are inhibited through the inhibition of upstream JAK protein. Therefore, JAK/STAT are potential therapeutic target in autoimmune and inflammatory diseases^70^. So, the amelioration of EAE by NTG-A-009 is due to modulation of JAK/STAT pathway (Supplementary Fig. S4).

We employed DSS induced colitis model in mice to find anti-inflammatory efficacy of NTG-A-009 on gastrointestinal disease. DSS is widely used chemical model in mice which resemble with IBD in human characterized by diarrhea, bloody feces, weight loss, colon shortening, and mucosal ulceration^71^. In this model leukocytes including lymphocytes, neutrophils, and macrophages have been found to infiltrate inflamed cells^71^. In DSS induced colitis the accumulation of T cells were found in inflamed colon which is similar to what is found in IBD patients^72^. This is in agreement with our findings that we showed higher accumulation of CD4^+^ and CD8^+^ T cells in colon which suggest the potential role of T cells in IBD pathogenesis. Th1 cells producing IFN-γ and Th17 cells producing IL-17A, IL-17F, IL-21 and IL-22 are found as the important mediators of inflammatory bowel disease (IBD)^73^. IBD is associated with aberrant Th1 responses and higher level of IFN-γ^74^. Th1 transcription factor T-bet is found to be important for the development of Th1 driven colitis which may be due to the higher level of IFN-γ in colitis^75^. It is found that Th17 associated cytokines are highly expressed in IBD. Tissue biopsies from inflamed colon showed high levels of Th17 and Th17 cells driven cytokines which suggest the involvement of Th17 in intestinal inflammation^76^. Consistent with above findings, *in vitro* and *in vivo* inhibition of Th1 and Th17 cells by NTG-A-009 encouraged us to choose DSS induced colitis model in mice. The disruption of mucus barrier by DSS allows the penetration of bacteria to underlying immune cells^72^. This leads to release of pro inflammatory cytokines and CD4^+^ T cells effector subsets^77^. Consistent with these findings, our results further boost the assumption that Th1 and Th17 are critical in the pathogenesis of colitis. So, the amelioration of DSS induced histological damage and improvement in body weight, colon length is due to the inhibition in Th1 and Th17 cells by NTG-A-009 treatment. Shorter treatment period with NTG-A-009 is found to be effective in ameliorating DSS. It is also noteworthy that in several studies the doses which are found to be effective for acute model of colitis were significantly high (100/500 mg/kg)^73,78^ but we have used minimal dose of NTG-A-009 (2 mg/kg/day) which is equally effective in attenuating colitis. The low dose regimen and short term treatment with NTG-A-009 in treating colitis make it potent compound for the treatment of colitis. However, DSS induced colitis and EAE are not clinically relevant murine models but multiple studies have shown that Th1 and Th17 are common mediators for colitis and EAE^9,73^. So, the inhibition of Th1 and Th17 cells differentiation by NTG-A-009 led us to perform EAE and DSS colitis as *in vivo* animal models.

In conclusion our findings strongly suggest that NTG-A-009 is effective for the treatment of EAE and DSS induced colitis via the suppression of Th1 and Th17 cells. We revealed the novel anti-inflammatory property of NTG-A-009 by its efficacy in treatment of autoimmune diseases which could be genesis for development of immune modulatory therapies.

## Materials and Methods

### Mice

C57BL/6 mice and OT-II mice were purchased from Jackson Laboratory and housed under specific pathogen free conditions at the Yeungnam University animal care center. All animal experimental protocols were approved and overseen by the Institutional Animal Care and Use Committee at Yeungnam University. Animals care and use were in accordance with the guidelines for care and use of laboratory animals.

### Flow Cytometry

For intracellular cytokine staining, cells were stimulated for 4 h in complete medium with phorbol 12-myristate 13-acetate (PMA) (50 ng/ml) (Sigma) and ionomycin (750 ng/mL) (Calbiochem) with protein transport inhibitors, GolgiStop (BD Biosciences) and incubated in a cell incubator with 5% CO_2_ at 37 °C. Cell surface staining was done in 1× PBS with FITC-conjugated anti-CD4 (GK1.5; Biolegend), PE-Cyanine7-conjugated anti-B220 (RA3-6B2; Biolegend), APC-conjugated anti-CD3ε (145-2C11 Biolegned), PE-Cyanine7-conjugated anti-CD8a (53 ± 6.7; Biolegend), PE-conjugated CXCR3 (CXCR3-173 Biolegend), PE-conjugated CCR6 (29-2L17 Biolegend). Cells were fixed and permeabilized using fixation and permeabilization buffer (BD bioscience) according to manufacturer’s instructions. For Foxp3 staining, cells were fixed and permeabilized using fixation and permeabilization buffer (ebioscience) according to manufacturer’s instructions. Antibodies used for cell staining included: anti-IFN-γ (PE, XMG; Biolegend), anti-IL-17-A (APC, TC11-18H10.1; Biolegend), anti-CD25 (PE, PC61; Biolegend), and anti-Foxp3 (APC, FJK-16s; eBioscience). Antibodies against phospho-JAK1 (Y1022/1023; Cell signaling), phospho-JAK2 (Y1007/1008; Cell signaling), phospho-STAT1 (Tyr701; Cell signaling), phopho-STAT3 (Tyr705; Cell signaling) and phospho-STAT4 (Tyr693; Cell signaling) used for immunoblot and flow cytometry were from BD Biosciences. Samples were run with BD FACS Verse flow cytometer (BD Biosciences), and data analysis was performed using FlowJo software.

### CD4^+^ T cell isolation, culture and *in vitro* differentiation

Naïve CD4^+^ T cells from the spleen and lymph nodes of 8–12 weeks C57BL/6 mice were purified by positive selection using CD4 conjugated magnetic beads (MiltenyiBiotec). CD4^+^ T cells (2 × 10^5^/well) were cultured *in vitro* for 3 days (37 °C, 5% CO_2_) on flat- bottom 96- well plates coated with anti-CD3 (5 μg/ml) (145-2C11; Biolegend) and Anti-CD28 (1 μg/mL) (37.51; eBioscience) in the presence of tofacitinib, triamcinolone or NTG-A-009. OVA-specific naïve CD4^+^ T cells from spleen and lymph nodes of OT-II mice were purified by positive selection with CD4-conjugated magnetic beads (MiltenyiBiotec), for antigen specific Th cell differentiation. CD4^+^ T cells (2 × 10^5^/well) and irradiated APCs (1 × 10^5^/well) were co-cultured in the presence of CD4^+^ T cells and were induced to differentiate in to Th1 cells under the supplementation with IL-12 (10 ng/mL) (Biolegend) and anti-IL4 (5 μg/mL) (Biolegend); as a blocking antibody. For Th17 differentiation, TGF-β1 (1 ng/mL) (R&D system), IL-6 (10 ng/mL) (R&D system) plus anti-IL-4 (5 μg/mL) (Biolegend) and anti- IFN-γ (5 μg/mL) (Biolegend); for T_reg_ differentiation, IL-2 (10 ng/ml) (Biolegend), TGF-β1 (10 ng/mL) (R&D system) plus anti-IL-4 (5 μg/mL) (Biolegend) and anti- IFN-γ (5 μg/mL) (Biolegend) and cultured in a cell incubator with 5% CO_2_ at 37 °C for 72 h.

### T cell proliferation assay

MACS- purified naïve CD4^+^ T cell isolated from spleen and lymph nodes of C57BL/6 mice were labelled with 5, 6-carboxyfluorescein diacetate N-succinimidyl ester (CFSE, eBioscience). Labelled cells were stimulated with plate bound anti-CD3 (5 μg/mL) and anti-CD-28 (1 μg/mL) in the presence of DMSO or NTG-A-009 in Th1 conditions (IL-12, 10 ng/mL plus anti-IL-4, 5 μg/mL) and Th17 differentiation conditions (IL-6, 10 ng/mL, TGF-β, 1 ng/ml plus anti-INF-γ and anti-IL-4, each at 5 μg/mL). Proliferation of cell was assessed after 72 h using flow cytometry. For Ki67 detection, isolated naïve CD4^+^ T cells were cultured under Th1 differentiation condition and stained with PE conjugated Ki-67 (Biolegend) after 72 h of culture. Proliferation of cell was assessed by flow cytometry. For the labeling of 5-bromo-2′-deoxyuridine (BrdU), naïve CD4^+^ T cells were isolated from spleen and lymph nodes from 8–12 weeks C57BL/6 mice and the cells were cultured under Th1 differentiation condition along with BrdU (10 μM). After 72 h of incubation, cells were stained with APC-conjugated BrdU as accordance to manufacturer’s protocol (BD Bio-sciences) in BrdU kit and proliferation of cells was assessed using flow cytometry.

### Apoptosis Assay

Naïve CD4^+^ T cells were isolated from the spleen and lymph nodes of 8–12 weeks C57BL/6 mice and purified by positive selection using CD4-conjugated magnetic beads (MiltenyiBiotec). Isolated CD4^+^ T cells were cultured for 72 h on 96 well plates coated with anti-CD3 (5 μg/ml) and Anti-CD28 (1 μg/mL) with or without NTG-A-009 under Th1 condition (IL-12, 10 ng/ml plus anti-IL-4, 5 μg/mL). Cells were then stained with Annexin V-APC and Propidium Iodide- PE as accordance to manufacturer’s protocol and analyzed by using flow cytometry.

### MTS assay

Splenocytes were isolated from normal C57BL/6 mice. 2 × 10^5^ cells/well were cultured in 96 well plate with RPMI 1640 medium containing 10% FBS and 1% penicillin and streptomycin in the presence of different concentration of NTG-A-009, Tofacitinib or Triamcinolone. Cells were incubated at 37 °C in 5% CO_2_ for 72 hours. 20 μl MTS reagent (Promega) was added per well and incubated for 2 hour. After incubation, absorbance was taken at 490 nm.

### CFA and OVA injection

Equal volume of complete Freund’s adjuvant (CFA, Chondrex Inc.) and ovaalbumin (OVA, Genemed synthesis Inc.) were mixed and injected intraperitoneally in C57BL/6 mice. NTG-A-009 (2 mg/kg/day) was injected in every other day for 6 days. Mice treated with PBS were considered as control group. At day 7, splenocytes were prepared and stimulated with phorbol 12-myristate 13-acetate (PMA) (50 ng/ml) (Sigma) and ionomycin (750 ng/mL) (Calbiochem) and GolgiStop (BD Biosciences) for 4 hours and stained with antibodies against IFN-γ and IL-17A. Results were then analyzed by Flow cytometry.

### Cytokine binding assay

Naïve CD4^+^ T cells isolated from the spleen and lymph nodes of 8–12 weeks C57BL/6 mice were cultured under Th1 and Th17 condition with or without NTG-A-009 for 3 days (37 °C, 5% CO_2_) on flat- bottom 96 well plates coated with anti-CD3 (5 μg/ml) and anti-CD28 (1 μg/mL). Cells were then washed and rested for 24 h in complete media containing 10% FBS followed by stimulation with phorbol 12-myristate 13-acetate (PMA) (50 ng/ml), ionomycin (750 ng/mL) for next 24 h. The quantification of harvested cells was done by using cytokine binding assay kit (740005, Biolegend) through flow cytometry.

### EAE induction and Treatment

Female C57BL/6 mice of 8–12 weeks old were immunized subcutaneously with synthetic peptide derived from myelin oligodendrocyte glycoprotein (MOG_35–55_) peptide (MEVGWYRSPFSRVVHLYRNGK, AnaSpec). Immunization was performed by mixing MOG_35–55_ peptide in CFA containing 10 mg/ml heat-killed H37Ra, strain of *Mycobacterium tuberculosis* (Difco Laboratories). Mice were immunized intraperitoneally with 250 ng pertussis toxin (List Biological Laboratories) on the day of MOG injection and then again 48 hours later. The examination of Mice was done daily and disease severity was scored using standard clinical score: 0- no obvious clinical change, 0.5- limp tail, 1- limp tail with no upright tail function, 1.5- wobbly gait, 2- flaccid tail with partial hind limb paralysis, 3- complete paralysis of hind limb, 4- moribund state, 5- dead. For EAE treatment, DMSO (as a control, Sigma-Aldrich) or NTG-A-009 (2 mg/kg/day) diluted in PBS and was administered intraperitoneally at day 0 and continued for every other day.

### DSS induced colitis

Colitis was induced in C57BL/6 mice by oral feeding of 2.5% (W/V) commercial detran sulfate sodium (MW 36–50 kDa, MP, Biomedicals, USA) in drinking water for 6 days. NTG-A-009 or water was orally administered every day till the end of the experiment. Only water treated group was taken as a control. Once the colitis was induced, the body weight and stool condition were analyzed on daily basis. The severity of the disease was analyzed by comparative measurement of percentage of weight loss and intestinal bleeding. The following scoring system can be used for the clinical assessment of stool, 0- normal stool with no blood, 1- slightly bloody with soft stool, 2- traces of blood with very soft stool, 3- visible rectal bleeding with watery stool.

### Histopathology

For EAE, Brain and spinal cord were obtained from control and NTG-A-009 treated mice and fixed immediately in 4% paraformaldehyde. 5–10 µm section of brain and spinal cord were stained with Hematoxylin and Eosin (H&E) staining for inflammation analysis and Luxol fast blue (LFB) for the analysis of degree of demyelination. For LFB staining, three free floating transverse section of spinal cord was taken and dried overnight on the subbed slide. Then the sections were briefly hydrated in 70% ethyl alcohol followed by incubation in LFB solution (Acros Organics) for 16 hour at 50 °C in water bath. After incubation, excess staining was removed and differentiation dipping was done in lithium carbonate solution (0.05% w/v) and later with 70% ethyl alcohol. These sections were then dehydrated with 95% and 100% ethyl alcohol successively. Finally the sections were cleared in xylene and images were observed under light microscope (Olympus BX41, Japan). For quantification, three transverse sections from thoracic lumber region were taken and 100X microscopic fields within white matter of the spinal cord were captured. Symmetrical sections from lateral funiculus part of the spinal cord with an equal distance covered by LFB stain were used for quantification purpose using ImageJ 1.48 v software.

For histological analysis of DSS induced colitis, the colon was separated from cecum and flashed in cold PBS for the removal of feces and blood. Small section of colon was immediately fixed in 4% paraformaldehyde and then stained with hematoxylin and eosin. The tissue pathology was determined by the inflammatory cell infiltration, presence of epithelial cell damage, crypt loss and reduction of goblet cells. Following parameters were used for the calculation of histological score: 0- none, 1- minimal loss of goblet cells, 2- extensive loss of goblet cells, 3- minimal loss of crypts and extensive loss of goblet cells, 4- extensive loss of crypts. For infiltration, 0- none, 1- infiltrate around crypt base, 2- infiltrate in muscularis mucosa, 3- extensive infiltrate in muscularis mucosa, 4- infiltration of submucosa.

### Isolation of lymphocyte from colonic lamina propria

Colons were collected and washed with PBS for the removal of feces. For the removal of epithelium, washed colons were incubated by gentle shaking for 30 min with calcium and magnesium free HBSS containing 1 mM EDTA. The colons were then sliced in to small pieces and resuspended in the digestion media containing RPMI 1640, 0.5 mg/ml collagenase D (Sigma) and 10 µg/ml DNase I for 1 hour with gentle shaking at 37 °C. After digestion, tissues were filtered through 40 µm cell strainer and washed with RPMI 1640 contained with EDTA. Cells were then resuspended with 40% percoll overlaid on 70% percoll. Cells collected from the interface of 40% and 70% gradient after centrifugation was the lamina propira lymphocytes (LPLs). Isolated lymphocytes were stimulated with phorbol 12-myristate 13-acetate (PMA) (50 ng/ml) (Sigma) and ionomycin (750 ng/mL) (Calbiochem) with protein transport inhibitors, GolgiStop (BD Biosciences) for 4 hours and stained with antibodies against IFNγ and IL-17A. Results were then analyzed by Flow cytometry.

### Mononuclear cell purification

For the purification of infiltrating mononuclear cells (MNCs) from brain and spinal cord, peripheral blood from mice was removed from heart by perfusion with PBS, single cell was prepared and MNCs were purified using percoll (GE Healthcare life sciences, South Korea) gradient (70%/30%).

### Transfection

CD4^+^ T cells were seeded in 60 mm disc at a density of 1 × 10^6^ cells/well and incubated overnight. Cells were then transfected with an STAT3 (pcDNA3-STAT3-Y705F, addgene plasmid) and STAT1 expressing plasmid (STAT1 alpha Y701F pRc/CMV, addgene plasmid) using fugene HD transfection reagent (Promega Madison, USA) as described by manufacturer protocol. After 48 hour of transfection the cells were treated with NTG-A-009 for 24 hour. The expression of phosphorylated and total form of STAT1 and STAT3 was analyzed by western blotting.

### Immunoblot analysis

Naïve CD4^+^ T cells were isolated from spleen and lymph nodes of 8–12 weeks C57BL/6 mice and purified by positive selection using CD4-conjugated magnetic beads (MiltenyiBiotec). The cells were then cultured for 72 h under Th1 and Th17 condition with or without NTG-A-009. After 72 h, cells were further stimulated with anti-CD3 and anti-CD28 for 30 min and then harvested.

For EAE, splenocytes from EAE control and NTG-A-009 treated mice were stimulated with MOG (200 µg/ml) for overnight. Protein sample was prepared from harvested cells using RIPA buffer containing protease and phosphatase inhibitors. Protein content was quantified using BCA protein assay reagent and separated by SDS-PAGE and blotted in to nitrocellulose membrane for 1 h followed by blocking for 1 h with 5% bovine serum albumin in Tris-buffered saline (TBS) with 0.1% Tween-20 for the analysis of both phosphorylated and total form. The blots were then probed overnight with primary monoclonal antibody at 4 °C. Next day blots were washed with TBST and incubated with secondary antibody for 1 h at temperature followed by band detection using ECL reagent kit (Pierce) under chemiluminescent image analyzer. β- actin antibody was used as a loading control.

### Total RNA isolation and quantitative real time PCR

Isolation of total RNA was performed from cell pellets using ReliaPrep^TM^ RNA cell Miniprep system (Promega, corporation, WI, USA) followed by cDNA synthesis using Goscript Reverse Transcription system (# A5001, Promega Corporation, WI, USA). QuantiTect SYBR Green PCR kit (Qiagen) was used for analysis of mRNA expression of each gene. The sequence of the primer used were: Il12a, 5′-CCACCC TT

GCCCTCCTAAAC-3′ and 5′-GGCAGCTCCCTCTTGTTGTG-3′; Il27, 5′-CTCTGCTTCCTC

GCTACCAC-3′ and 5′-GGGGCAGCTTCTTTTCTTCT-3′; Il23, 5′-AAGTTCTCTCCTCTT

CCCTGTCGC-3′ and 5′-TCTTGTGGAGCAGCAGATGTGAG-3′;Il6,5′ TATGAAGTTCCTCTCTGCAAGAGA-3′ and 5′- TAGGGAAGGCCGTGGTT-3′; Il1b, 5′-AAGGAGAACCAAGCAACGACAAAA-3′ and 5′-TGGGGAACTCTGCAGACTCAAACT 3′; Il10, 5′-ATAACTGCACCCACTTCCCAGTC-3′ and 5′CCCAAGTAACCCTTAAAGTCCTGC-3′;β-actin, 5′-TGTCCACCTTCCAGCAGATGT-3′ and 5′AGCTCAGTAACAGTCCGCCTAGA.

### Statistical analysis

The statistical significance was analyzed using student *t-* test or ANOVA wherever appropriate (Graph Pad Prism 5.0 software, San Diego, CA, USA). *P*-value < 0.05 was considered as statistically significant. Data were the representative of three independent experiments and presented as mean ± S.E.M. The analyses of FACS data were done using Flowjo software.

## Electronic supplementary material

supplementary information
