# Supplementary material for: Amelioration of Experimental autoimmune encephalomyelitis and DSS induced colitis by NTG-A-009 through the inhibition of Th1 and Th17 cells differentiation
Source: Sci Rep. 2018 May 17;8:7799. doi: 10.1038/s41598-018-26088-y (PMC5958108; doi:10.1038/s41598-018-26088-y)
Supplement: Supplementary file 1 — supplementary information [file 41598_2018_26088_MOESM1_ESM.pdf]

Title:

Amelioration of Experimental autoimmune encephalomyelitis and DSS induced colitis by NTG-A-009 through the inhibition of Th1 and Th17 cells differentiation.

Authors:

Suman Acharya[1], Maheshwor Timilshina[1], Liyuan Jiang[1], Sabita Neupane[1], Dong-Young Choi[1], Sang Won Park[2], Sang Yeul Lee[2], Byeong-Seon Jeong[1], Jung-Ae Kim[1], Tae-gyu Nam[2]*, Jae-Hoon Chang[1]*

[1]College of Pharmacy, Yeungnam University, Gyeongsan 38541, Republic of Korea
[2]Department of Pharmacy and Institute of Pharmaceutical Science and Technology, Hanyang University, Ansan 15588, Republic of Korea
*Correspondence to: Jae-Hoon Chang and Tae-gyu Nam
Jae-Hoon Chang: Email: jchang@yu.ac.kr; Tel: +82-53-810-2833; Fax: +82-53-810-4654
Tae-gyu Nam: Email: tnam@hanyang.ac.kr, Tel: +82-31-400-5807; Fax: +82-31-400-5958

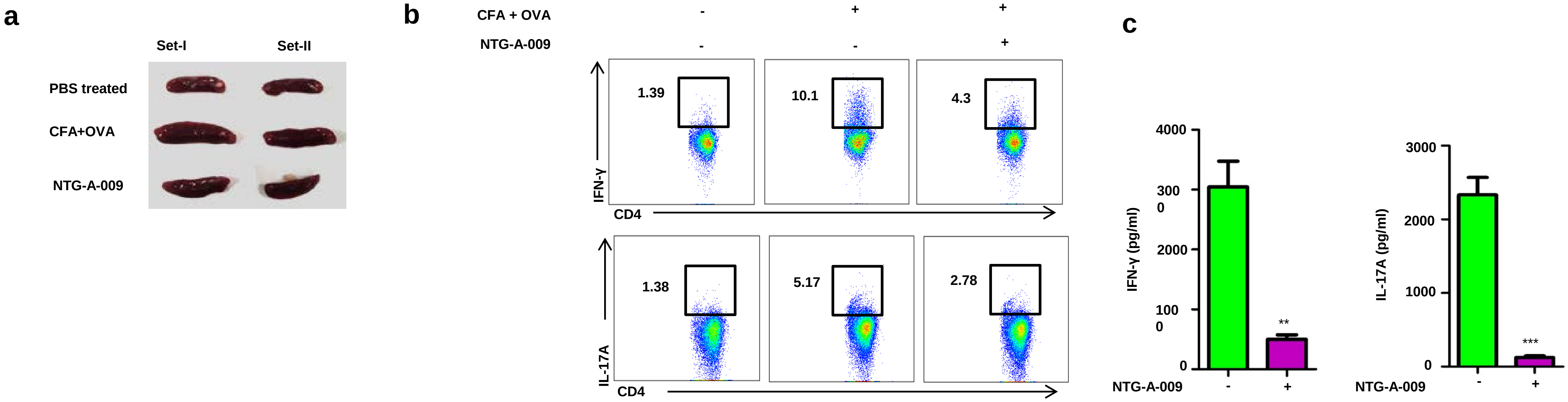

Supplementary information 1- Inflammation suppression by NTG-A-009 in CFA and OVA injected mice. (a) 8-12 weeks C57BL/6 mice were intraperitoneally immunized with 200ul of CFA and OVA mixture for the induction of acute inflammation. PBS or NTG-A-009 (2 mg/kg/day) was administered intraperitoneally in every other day. Images of spleens from PBS, CFA/OVA and NTG-A-009 treated mice at day 7 were compared. **(b)** Spleens and lymph nodes were restimulated for 4 hour with phorbol 12-myristate 13-acetate and ionomycin and the percentage of Th1 and Th17 was analyzed by FACS through intracellular staining of IFN-γ and IL-17A. (**c**)**-** The quantification of IFN-γ and IL-17A was assessed by cytokine binding assay as described in materials and methods. Data represent three independent experiments with similar results. Mean ± SEM of the triplicates are shown **P< 0.01; ***P< 0.001.

a

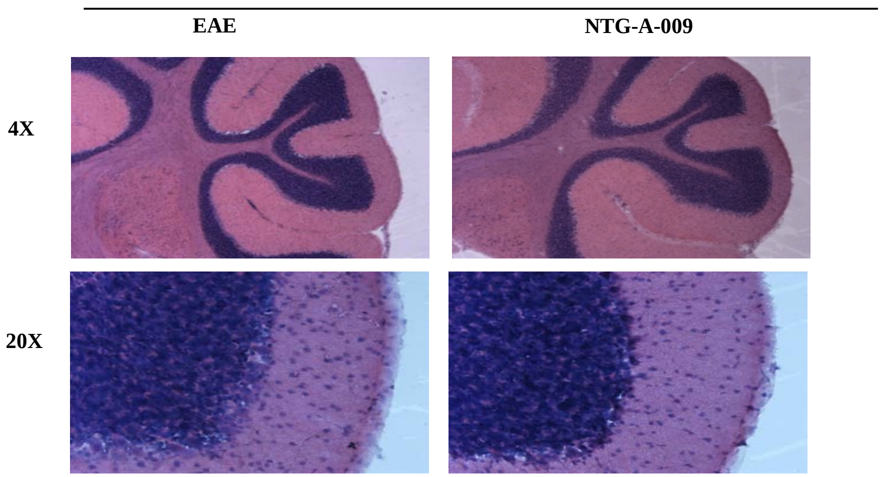

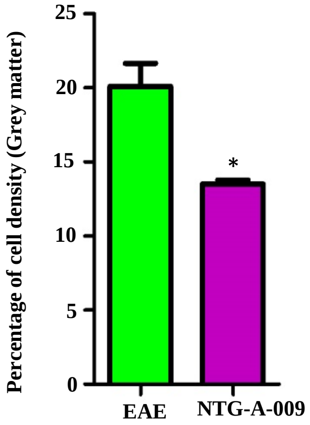

Supplementary information 2- NTG-A-009 reduces the infiltration of inflammatory cells in brain. (a) Section of the brain obtained from the EAE control and NTG-A-009 treated mice was analyzed for inflammation by H & E staining. Bar diagram denotes the total cell density. Data are the representative of three independent experiments. Mean ± SEM of the triplicates are shown *$p < 0.05$*

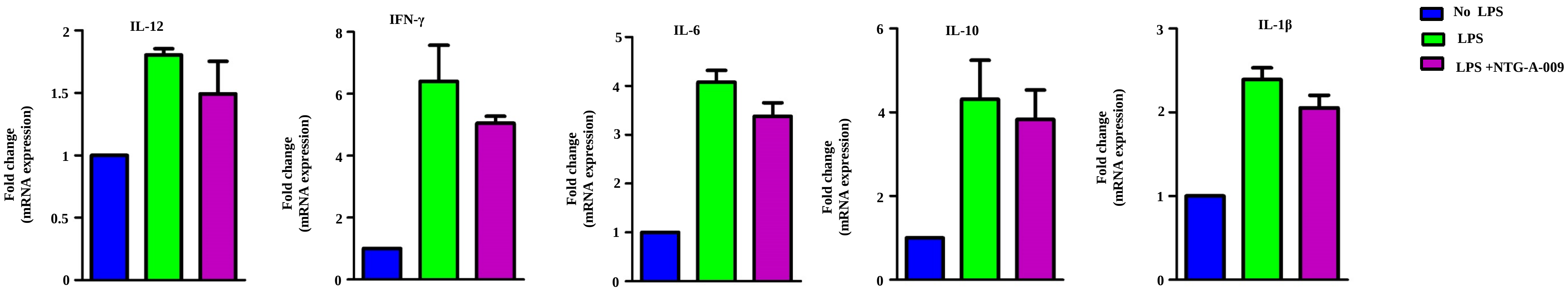

Supplementary information 3- NTG-A-009 has no inhibitory effect on bone marrow derived dendritic cells. Bone marrow was isolated from tibias and femur of 6-8 weeks female C57BL/6 mice and co-cultured with 20 ng/ml recombinant granulocyte macrophage colony stimulation factor (GM-CSF). At day 8 non-adherent cells were collected and cultured with NTG-A-009 along with 200 ng/ml lipopolysaccharides (LPS) for 4 hours. RNA was prepared followed by cDNA preparation **A-** mRNA expression of related cytokines was shown. Data are the representative of three independent experiments. Mean ± SEM of the triplicates are shown

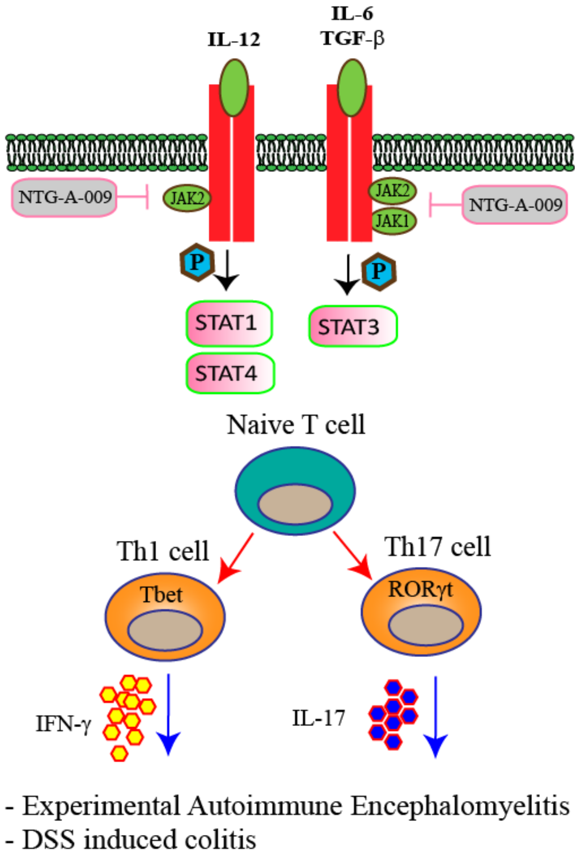

Supplementary information 4- Proposed molecular mechanisms of NTG-A-009. IL-12 activates STAT4 for Th1 differentiation through the activation of its upstream protein JAK2 while IL-6 and TGF- β induces STAT3 for the differentiation and function of Th17 through the activation of JAK1/JAK2.

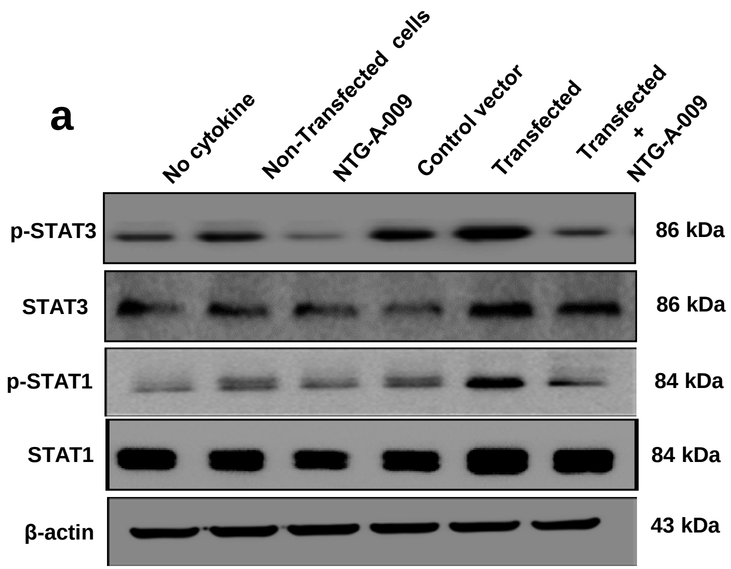

Supplementary information 5- Effects of NTG-A-009 on overexpressed STAT1 and STAT3 proteins. (a) CD4^{+} T cells were transfected with an STAT1 and STAT3 expressing plasmid. After 48 hour of transfection, the cells were treated with NTG-A-009 for 24 hour. The expression of phosphorylated and total form of STAT1 and STAT3 was analyzed by western blotting. Data are the representative of three independent experiments.

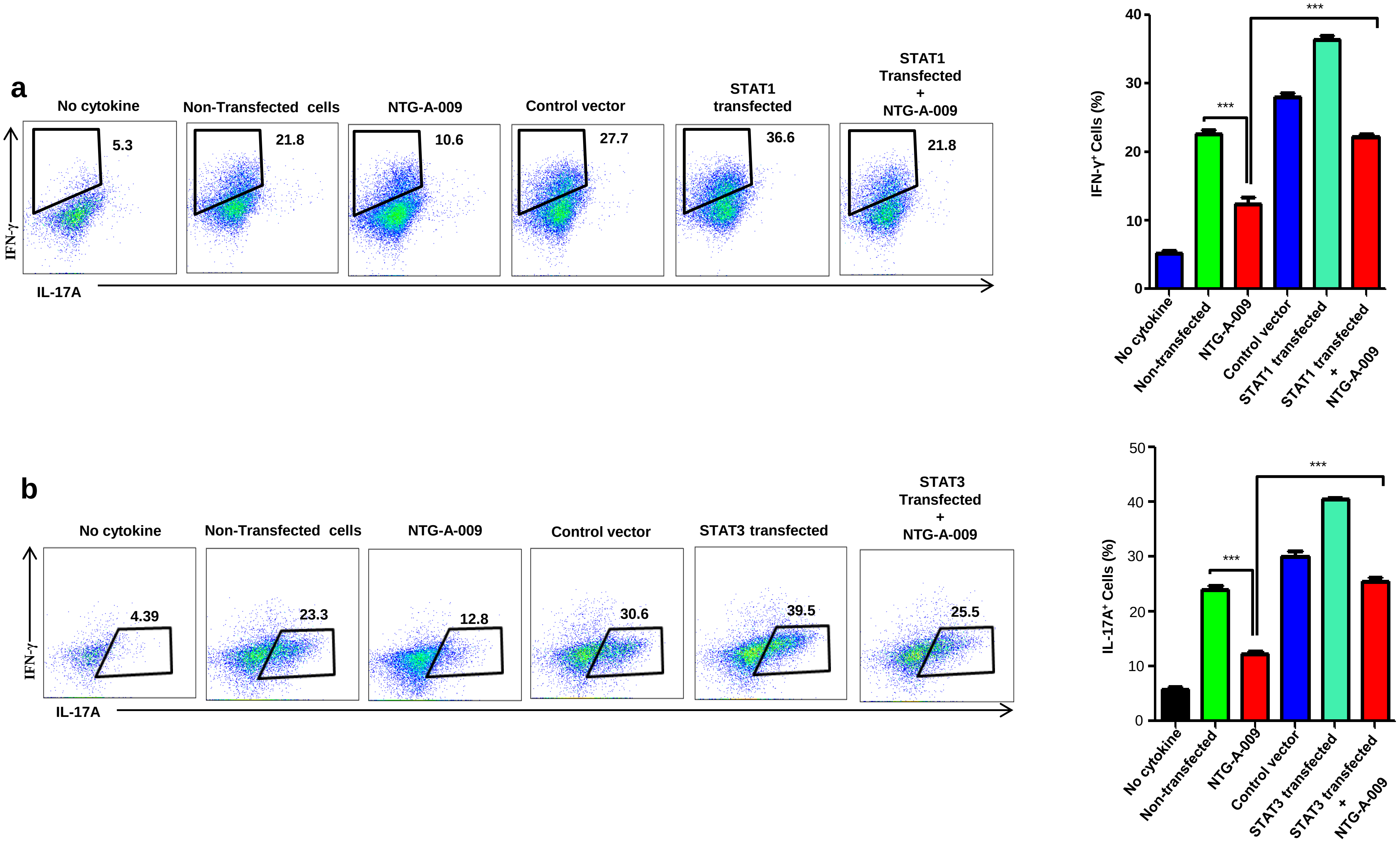

Supplementary information 6- Effect of NTG-A-009 on Th1 and Th17 differentiation from STAT1 and STAT3 overexpressed cells. Transfected and non-transfected CD4 T cells were stimulated with anti-CD3 (1μg/ml) and anti-CD28 (1μg/ml) in the presence or absence of NTG-A-009 under Th1 and Th17 differentiation condition. **(a,b)** The percentage of IFN-γ^+ Th1 **(a)** and IL-17A^+ Th17 cells **(b)** was determined by flow cytometry. Data are the representative of three independent experiment. Mean ± SEM of the triplicates are shown ****P< 0.001*.

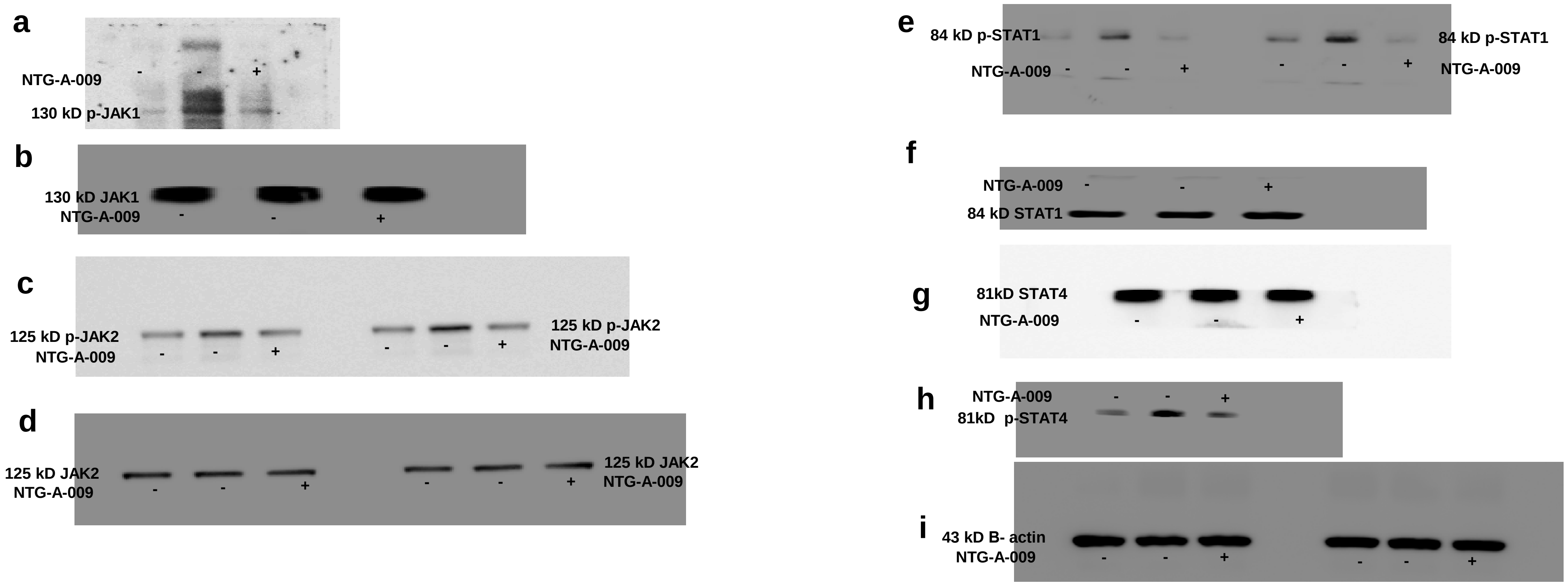

Supplementary information 7- uncropped scans of the western blot. Main figure 3(a) naïve CD4+ T cells cultured under Th1 condition in the presence or absence of NTG-A-009 and further stimulated with anti-CD3 and anti-CD28 and immunoblot was carried out. representative images of **(a, b)** phosphorylated and total form of JAK1 proteins, **(c- h)** phosphorylated and total form of JAK2, STAT1, STAT4 proteins respectively. (**i)** representative image of β-actin was shown.

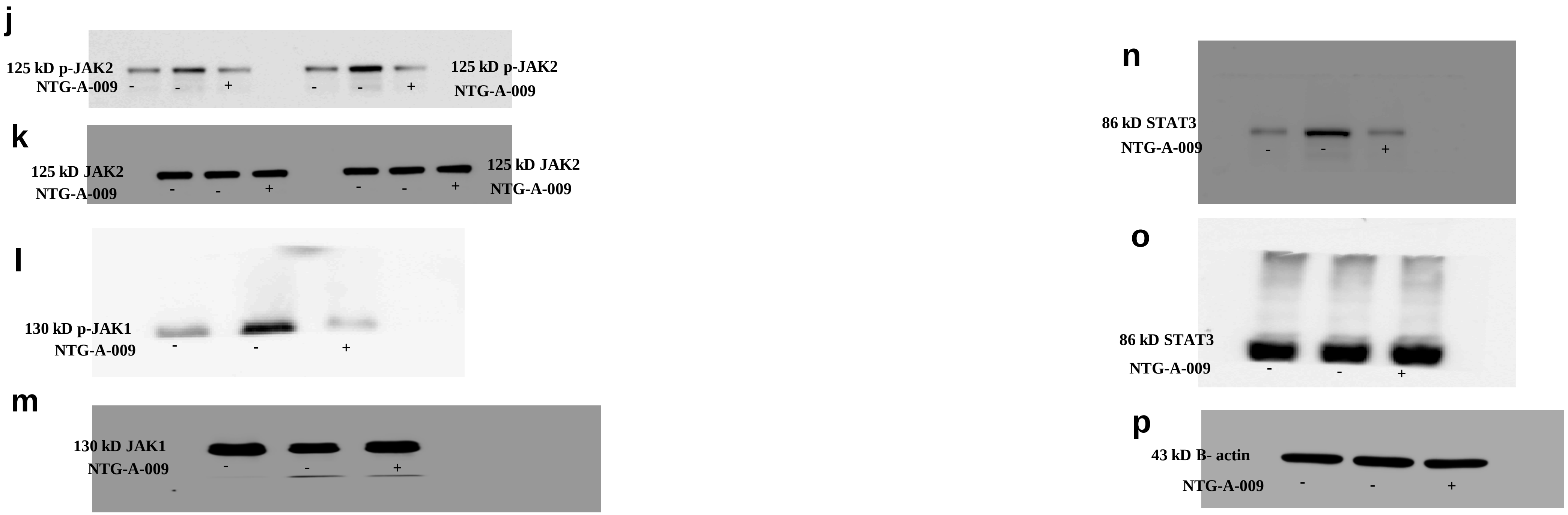

Supplementary information 8- uncropped scans of the western blot. Main figure 3(b) naïve CD4+ T cells cultured under Th17 condition in the presence or absence of NTG-A-009 and further stimulated with anti-CD3 and anti-CD28 and immunoblot was carried out. representative images of **(j,k)** phosphorylated and total form of JAK2 proteins, **(l-o)** phosphorylated and total form of JAK1 and STAT3 proteins respectively. **(i)** representative image of β-actin was detected by immunoblotting under Th17 condition.

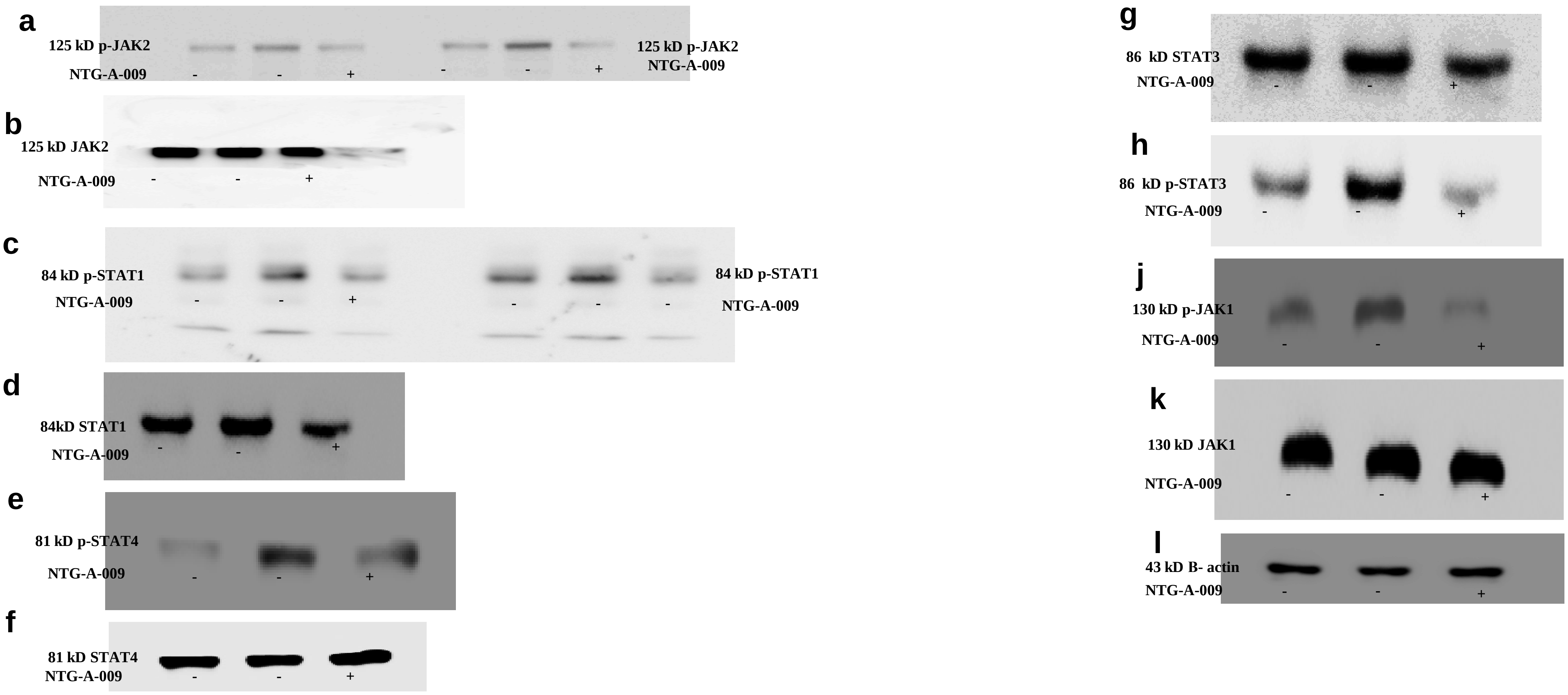

Supplementary information 9- uncropped scans of the western blot. Main figure 5 Splenocytes derived from the EAE and NTG-A-009 treated mice were further overnight restimulated with MOG $_{35\text{-}55}$ peptide and CD4^{+} T cells were analyzed. Immunoblotting was done for the expression of phosphorylated form of JAK/STAT proteins in JAJK/STAT signaling pathway. **(a-k)** Representative images of phosphorylated and total form of JAK2, STAT1, STAT4, STAT3, JAk1 were shown respectively. **(l)** image of β-actin that was used as loading control.

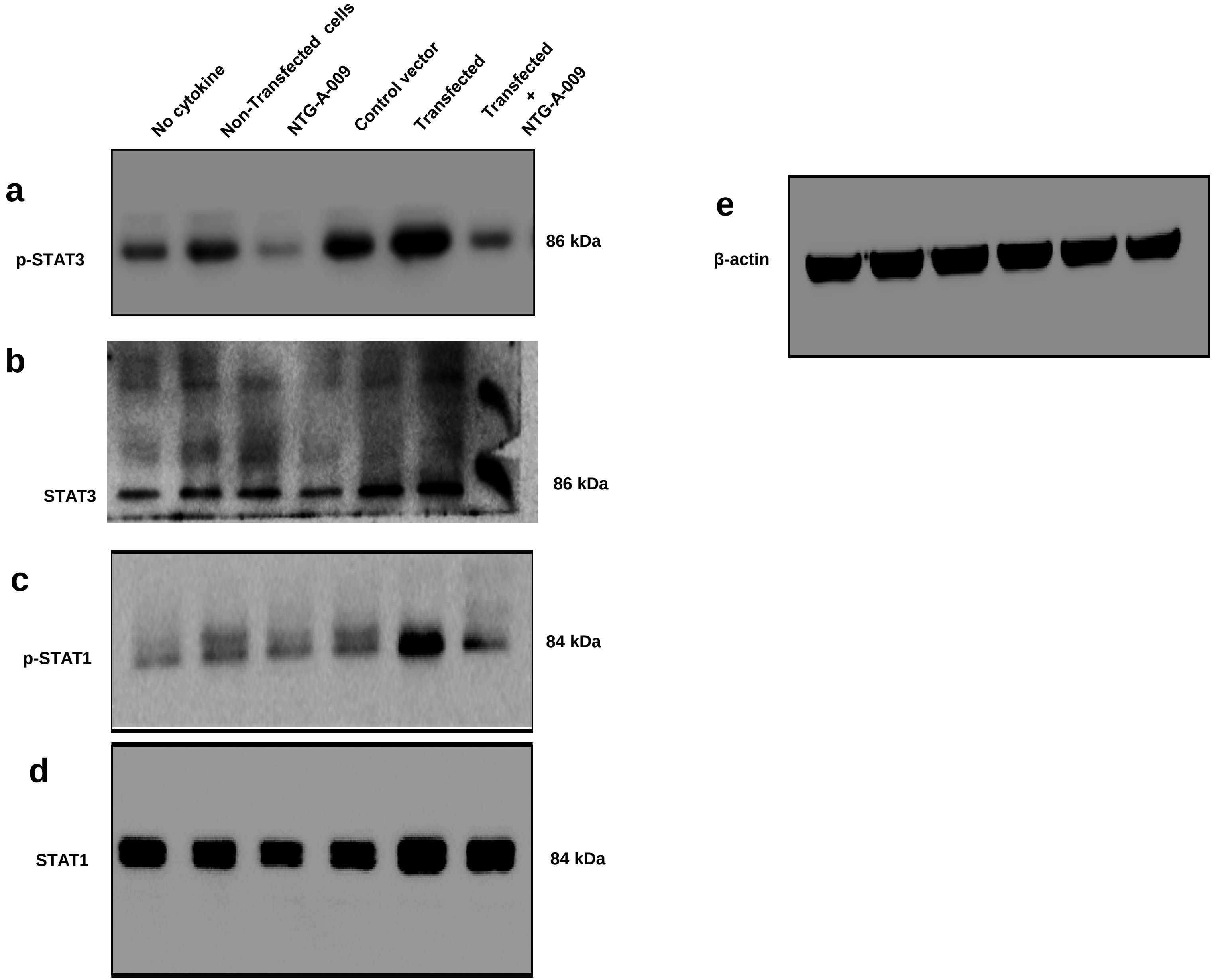

Supplementary information 10- uncropped scans of supplementary figure 5a . $CD4^+$ T cells were transfected with an STAT1 and STAT3 expressing plasmid. After 48 hour of transfection, the cells were treated with NTG-A-009 for 24 hour. **(a-e)** The expression of phosphorylated and total form of STAT1 and STAT3 was analyzed by western blotting. Data are the representative of three independent experiments.
